# A possible new activator of PI3K‐Huayu Qutan Recipe alleviates mitochondrial apoptosis in obesity rats with acute myocardial infarction

**DOI:** 10.1111/jcmm.17353

**Published:** 2022-05-13

**Authors:** He Tai, Yu‐jing Tong, Rui Yu, You Yu, Si‐cheng Yao, Ling‐bing Li, Ye Liu, Xiao‐zheng Cui, Jin‐song Kuang, Xian‐sheng Meng, Xiao‐lin Jiang

**Affiliations:** ^1^ 66473 School of Pharmacy Liaoning University of Traditional Chinese Medicine Dalian China; ^2^ Department of Internal Medicine Liaoning Provincial Corps Hospital of Chinese People's Armed Police Forces Shenyang China; ^3^ 85024 Department of Pediatrics Shengjing Hospital of China Medical University Shenyang China; ^4^ 66473 Science and Technology Branch Liaoning University of Traditional Chinese Medicine Shenyang China; ^5^ 66473 Key Laboratory of Ministry of Education for Traditional Chinese Medicine Viscera‐State Theory and Applications Liaoning University of Traditional Chinese Medicine Shenyang China; ^6^ Department of Graduate School China PLA General Hospital Beijing China; ^7^ Third Affiliated Hospital of Beijing University of Chinese Medicine Beijing China; ^8^ 12442 Cardiovascular Surgery School of Clinical Medicine Beijing Tsinghua Changgung Hospital Tsinghua University Beijing China; ^9^ Department of Endocrinology and Metabolism The Fourth People's Hospital of Shenyang Shenyang China; ^10^ Nephrology Laboratory The fourth of Affiliated Hospital of Guangzhou University of Traditional Chinese Medicine (Shenzhen Traditional Chinese Medicine Hospital) Guangzhou University of Traditional Chinese Medicine Shenzhen China

**Keywords:** acute myocardial infarction, apoptosis, Huayu Qutan Recipe, mitochondrial dysfunction, obesity, PI3K/Akt/Bad pathway

## Abstract

Obesity, which has unknown pathogenesis, can increase the frequency and seriousness of acute myocardial infarction (AMI). This study evaluated effect of Huayu Qutan Recipe (HQR) pretreatment on myocardial apoptosis induced by AMI by regulating mitochondrial function via PI3K/Akt/Bad pathway in rats with obesity. For in vivo experiments, 60 male rats were randomly divided into 6 groups: sham group, AMI group, AMI (obese) group, 4.5, 9.0 and 18.0 g/kg/d HQR groups. The models fed on HQR with different concentrations for 2 weeks before AMI. For in vitro experiments, the cardiomyocytes line (H9c2) was used. Cells were pretreated with palmitic acid (PA) for 24 h, then to build hypoxia model followed by HQR‐containing serum for 24 h. Related indicators were also detected. In vivo, HQR can lessen pathohistological damage and apoptosis after AMI. In addition, HQR improves blood fat levels, cardiac function, inflammatory factor, the balance of oxidation and antioxidation, as well as lessen infarction in rats with obesity after AMI. Meanwhile, HQR can diminish myocardial cell death by improving mitochondrial function via PI3K/Akt/Bad pathway activation. In vitro, HQR inhibited H9c2 cells apoptosis, improved mitochondrial function and activated the PI3K/Akt/Bad pathway, but effects can be peripeteiad by LY294002. Myocardial mitochondrial dysfunction occurs following AMI and can lead to myocardial apoptosis, which can be aggravated by obesity. HQR can relieve myocardial apoptosis by improving mitochondrial function via the PI3K/Akt/Bad pathway in rats with obesity.

## 1 INTRODUCTION

As a severe disease, acute myocardial infarction (AMI) is responsible for cardiac insufficiency and even cardiac structure impairment.^1^ Moreover, AMI is not only one of the most dangerous coronary heart diseases but is also an important factor for cardiac insufficiency; it can induce death in critical patients.^2^ A decline in contractility and a large increase in myocardial infarction are the major pathophysiological manifestations observed after AMI.^3^ In this context, this study proposes a new approach for preventing AMI and facilitating the treatment of AMI.

Increases in obesity, hyperuricemia, diabetes and hyperlipidaemia (HLP) are often accompanied by obesity, which can induce hypertension and insulin resistance. Both can be seen as chronic hyper‐inflammatory states.^4^ A significant predisposing factor HLP often occurs with AMI.^5^ After AMI, the infarct size can be increased by acute mixed HLP.^6^ HLP is a highly specific risk factor for cardiovascular disease.^7^, ^8^ The pathobiological value of HLP in AMI patients has yet to be determined. Mitochondrial dysfunction plays an important role in AMI.^9^, ^10^

In Asian countries, the treatment of heart disease, including angina, chronic heart failure and myocardial infarction, widely employs traditional Chinese medicine (TCM). As the Chinese herbal compound, Huayu Qutan Recipe (HQR) primarily consists 9 herbal compounds. Our previous study has shown that HQR regulates lipid metabolism via the SREBP‐2 signal pathway.^11^ However, studies on HQR myocardial protection in rats with obesity have rarely been reported. The current study focuses on evaluating AMI‐induced mitochondrial dysfunction to better reduce myocardial injury by using HQR, as well as to cross the gist between mitochondrial dysfunction and myocardial cell apoptosis in order to identify new therapeutic targets and examine the correlated underlying mechanisms.

## 2 MATERIALS AND METHODS

### 2.1 Chemicals and reagents

Huayu Qutan Recipe granules were provided by Sichuan New Green Pharmaceutical Co., Ltd in China. Huayu Qutan Recipe granules were dissolved in heated deionized water (60°C) to obtain stock solutions with different concentrations (0.45, 0.9 and 1.8 g/ml). The stock solutions were preserved in refrigerator (4°C).

### 2.2 Chemical component analysis

**FIGURE 1:**
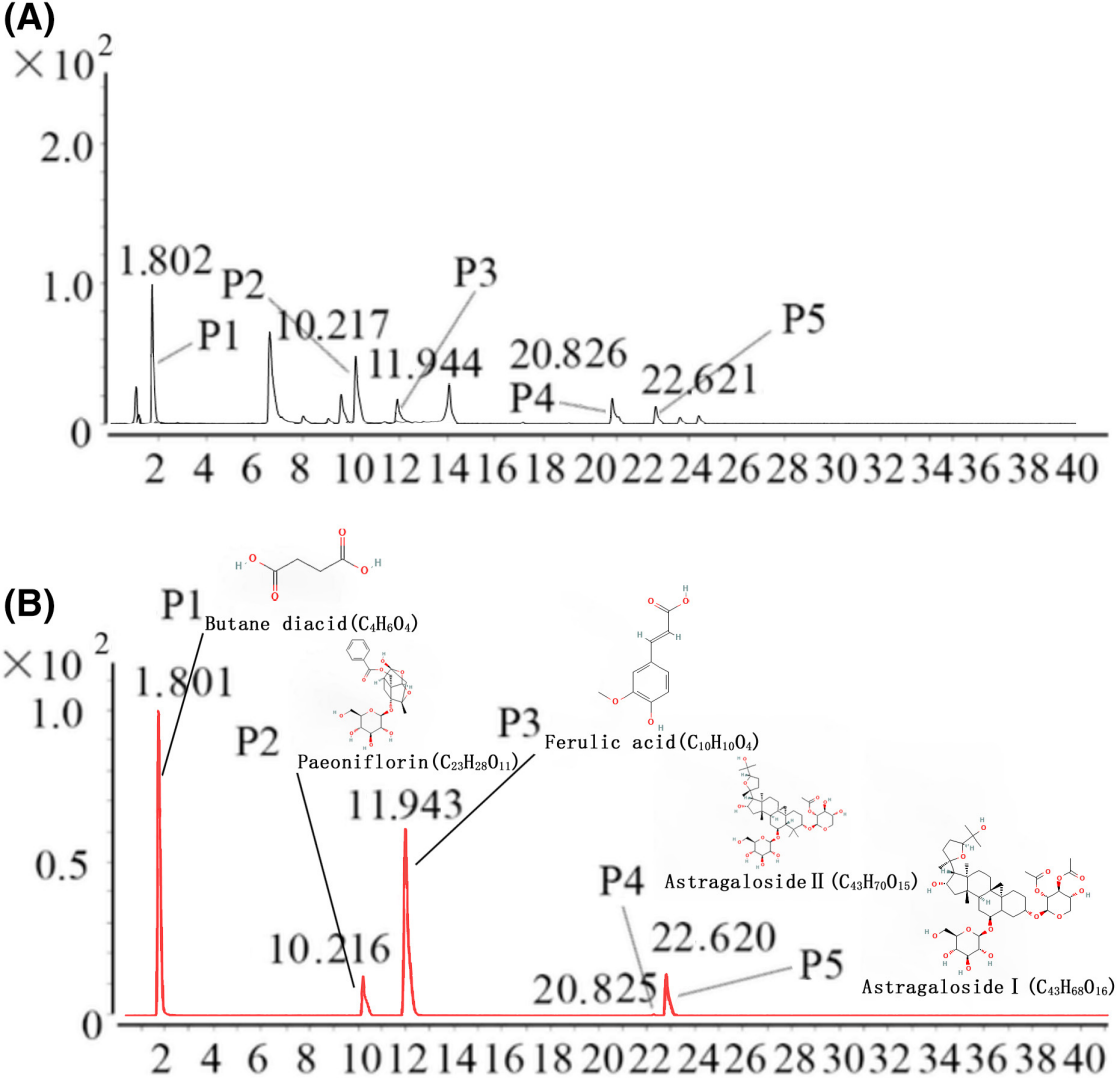
Extracted ion chromatograms of Huayu Qutan recipe (HQR). The extracted ion chromatograms of mixed standards (A); the extracted ion chromatograms of HQR sample: 1. Butane diacid (CAS: 110‐15‐6, 117.0193 M–H, 1.802 min); 2. Paeoniflorin (CAS: 23180‐57‐6, 479.1504 M–H, 10.217 min); 3. Ferulic acid (CAS: 537‐98‐4, 193.0491 M–H, 11.944 min); 4. Astragaloside Ⅱ (CAS: 91739‐01‐4, 819.4205 M+Na‐2H, 20.826 min); and 5. Astragaloside Ⅰ (CAS: 84680‐75‐1, 861.4299 M+Na‐2H, 22.621 min) (B)

Huayu Qutan Recipe was granted a national patent (Patent number: ZL200710010845.9). The compound contains 10 herbs: air‐dried roots of *Salvia miltiorrhiza* (family: Labiatae) (5 g); dried sclerotia of *Wolfiporia cocos* (family: Polyporaceae) (10 g); air‐dried roots and rhizomes of *Acorus tatarinowii*. (family: Araceae) (5 g); air‐dried roots of C*urcuma* (family: Zingiberaceae) (5 g); processed *Pinellia ternata* (family: Araceae) (5 g); a whole plate of air‐dried *Gynostemma pentaphyllum* (family: Cucurbitaceae) (15 g); air‐dried roots of *Codonopsis pilosula* (family: Campanulaceae) (25 g); air‐dried rhizomes and roots of *Ligusticum chuanxiong* Hort (family: Umbelliferae) (5 g); and air‐dried rhizomes and roots of *Astragalus membranaceus* (family: Papilionaceae [Leguminosae]) (25 g). The main components of HQR were analysed by quadrupole‐time‐of‐flight mass spectrometry (Q‐TOF MS/MS) coupled with high‐performance liquid chromatography (HPLC). The major components of HQR were authenticated using real standards. The status of chromatographic and mass spectrometry are shown in Figure 1.

### 2.3 Experimental animals

Male Sprague‐Dawley rats (aged 8 weeks and weighing 180–220 g) were used in the study. The rats were purchased from Changsheng (Liaoning) Biotechnology Co., Ltd. (SCXK (Liao) 2015‐0001). The rats were raised in coops under the following controlled conditions: humidity, of 45%–65%; temperature, 20°C ± 3°C; and dark/light cycle, 12 h/12 h (lights on 06:00 H). The rats had free access to a pellet diet and water.

A total of 60 male rats were divided into 6 groups, as follows: sham‐operated group, AMI group, AMI (obese) group, the groups fed with 4.5 g/kg/d HQR, 9.0 g/kg/d and 18.0 g/kg/d HQR. Each group included 10 rats. All rats in each group were fed with general maintenance feed for 2 weeks to conform to the controlled environment. The rats in the AMI and sham groups were raised on general maintenance feed for 8 weeks, whereas those in the other 4 groups were raised on a high‐fat diet (HFD). Three groups—the sham group, the AMI group and the AMI (obese) group—received intragastric infusions with deionized water. The other 3 groups were received intragastric infusions of HQR with different concentrations (4.5, 9.0 and 18.0 g/kg/d) for 2 weeks before AMI. The components of HFD were as follows: 13% fibre, 44% carbohydrate, 11% unsaturated fat, 25% total fat containing 18% protein, ash and other ingredients.^12^ The rats with a 30% increase in body weight indicated that the obesity model was successfully established and thus can be selected for further study.^13^

### 2.4 Surgical procedure

Rats were anaesthetized (isoflurane via inhalation anaesthesia), then to assess for anaesthetic effects, the rats were paw‐pinched and tail‐pinched. The AMI model was established as described in a previous report.^14^ A left thoracotomy through the fifth intercostal space was performed to completely reveal the heart. The left anterior descending branch of the heart was permanently ligated with a 6–0 silk suture.

### 2.5 Detecting blood fat, myocardial enzyme spectrum and inflammatory factor in serum

Blood was extracted from the abdominal aorta to detect the levels of blood fat (total cholesterol [TC]), low‐density lipid‐cholesterol [LDL‐C], triglyceride [TG], and high‐density lipid cholesterol [HDL‐C], myocardial enzyme spectrum (CK‐MB and cTNI) and inflammatory factors (TNF‐α and IL‐1β). They were examined following the instruction provided in the reagent kit.

### 2.6 Detecting cardiac function

Noninvasive transthoracic echocardiography (VisualSonics of Vevo2100) was employed to assess the left ventricle morphology and function of anaesthetized rats (Matrx VIP 3000). The mean included a two‐dimensional mode, including blood flow detection with the pulsed‐mode Doppler and the time‐motion ultrasound. The left ventricular end‐diastolic internal diameter (LVIDd) and left ventricular end‐systolic internal diameter (LVID), left ventricular fractional shortening (FS) and left ventricular ejection fraction (EF) were measured and calculated.^15^

### 2.7 Myocardial infarction area measurement

After successful AMI angioplasty, 5 ml of blood was drawn from the caudal vein, and 2 ml of Evans blue dye (0.5%) was injected into the carotid artery. After the dye was distributed to the whole‐heart tissue (out of the blood supply area of the anterior descending branch in the left coronary artery), the rat was anaesthetized, and the heart was quickly removed. The ischaemic area, infarct area and normal area were observed. After the heart was fixed with 4% paraformaldehyde and restained with 1% red tetrazolium for 15 min, the ischaemic part appeared red, the infarcted part turned white, and the normal part was blue. The degree of infarction was determined using the following formula: the degree of infarction = (infarct/ischaemic area) × 100%.

### 2.8 Histological evaluation of myocardial tissue via haematoxylin–eosin staining

Cutting myocardial tissues (non‐necrotic part) were sliced into thin sections with a thickness of 5 μm and then haematoxylin–eosin (HE)‐stained after paraffin embedding as described in a previous study.^16^ The myocardial tissues were infiltrated for 24 h with 4% paraformaldehyde and then diverted to 70% ethanol. The myocardial tissues were observed by light microscopy.^17^

The degree of myocardial damage was evaluated on a scale of 0 to 4, as follows: 0 (normal), preserved normal myocardial tissues; 1 (minor damage), localized necrosis and interstitial oedema; 2 (moderate damage), extensive myocardial cell necrosis and swelling; 3 (severe damage), neutrophil infiltration, compressed capillaries, and necrosis with contraction bands; and 4 (highly severe damage), compressed capillaries and haemorrhaging, neutrophil infiltration, and diffuse necrosis with contraction bands.^18^

### 2.9 SOD and MDA in myocardial tissue

The myocardial tissue was placed on an ice table, and the normal myocardial tissue (5–10 mg) around the necrotic area was removed. The myocardial tissue was weighed and then crushed with scissors and a homogenizer. The 5%–10% tissue homogenate (5–10 ml) was prepared by adding normal saline. In accordance with the instructions provided in the reagent kit, the tissue homogenate and reagent were mixed in a centrifuge tube and then cooled immediately at 95°C after being taken out (40 min). The supernatant was centrifuged for 10 min at 3500–4000 × *g*. The supernatant was taken out at 532 nm with 1 cm light diameter. The distilled water was zeroed, the absorbance was measured, and the malondialdehyde (MDA) was measured based on the absorbance value. The prepared tissue homogenate was mixed with corresponding reagents as specified in the instructions provided with the reagent kit, and the superoxide dismutase (SOD) was determined at 550 nm.

### 2.10 Observation of mitochondria by electron microscopy

After being thinly sliced (1 mm^3^ small volume), the myocardial tissues were collected immediately. The tissues were fixed in 2% glutaraldehyde in an environment at 4°C. They were washed with pH 7.4 phosphate‐buffered saline (0.1 mol/L) and then fixed in 1% osmium tetroxide by using 1% aqueous uranyl acetate to stain. The medium was embedded with capsules, and the specimens were placed on the slide for about 48 h (70°C). Alkaline lead citrate and uranyl acetate were used to stain the sections, which were then visualized by electron microscopy (Hitachi/H‐7650, Japan).

### 2.11 Using TUNEL to assess apoptosis in the myocardial tissues

Terminal deoxynucleotidyl transferase‐mediated dUTP nick end‐labelling (TUNEL) was conducted to evaluate apoptosis by using the *In Situ* Cell Death Detection Kit (Solarbio). Paraffin sections were used for TUNEL staining.^16^ After deparaffinization, the paraffin sections were incubated with 10 μg/ml of proteinase K and then rehydrated for 15 min. Fresh TUNEL reaction mixtures were added to sections, which were incubated for 60 min in darkness and at 37°C. After they were washed, the cell nuclei were stained with 0.1 μg/ml 4′,6‐diamidino‐2‐phenylindole (DAPI). The sections were analysed with a drop of PBS by fluorescence microscopy (Canon).

### 2.12 Caspase‐9/3 activity

Caspase‐9/3 activity in the myocardial tissues was detected using the fluorescent caspase‐specific substrate AcDEVD‐7‐pNA (Solarbio). Myocardial tissues (10 mg) were added to the reaction buffer and then incubated for 2 h at 37°C. The enzyme‐catalysed release was quantified using a fluorimeter (405 nm).

### 2.13 Preparation of the mitochondrial suspension

The rats were executed as described in a previous study.^19^ The heart was removed and placed in a breaker with a pH 7.4 ice‐cold isolated buffer (1 mM EDTA, 250 mM sucrose and 10 mM Tris‐HCl). After the tissue samples were trimmed, they were rinsed using a homogenizer in an isolation buffer and then taken out (50–100 mg). The entire process was performed at 4°C to maintain mitochondrial integrity. Centrifugation (700 × *g*) was conducted for 10 min, and the supernatant was again collected for centrifugation (7000 × *g*) for 10 min. The supernatant was then discarded. Resuspension was conducted to wash the mitochondrial pellets (5 ml isolated buffer) and centrifuge (7000 × *g*) then twice for 10 min. The clean mitochondrial solution was removed, and a mitochondrial preservation solution (KCl 100 mM, MgCl_2_ 2 mM, KH2PO_4_ 10 mM, sucrose 20 mM, EDTA 1 mM and HEPES 5 mM) was used as a maintenance solution to obtain a mitochondrial suspension (5–10 mg/ml protein) and then placed on ice for immediate use. The protein level of the mitochondrial suspension was detected with the bicinchoninic acid (BCA) reagent (Beyotime) to ensure that the protein level is in the 100–1000 μg/ml range. The mitochondrial suspension was used to detect the mitochondrial membrane potential (MMP),^19^ the mPTP opening,^20^ reactive oxygen species (ROS), damaged mtDNA,^21^ mitochondrial oxygen consumption rate,^22^ respiratory control rate (RCR),^22^ respiratory chain complex enzymes (Ⅰ, Ⅱ, Ⅲ, Ⅳ and Ⅴ) in mitochondria^23^ and adenosine triphosphate (ATP).^23^

### 2.14 RNA extraction and cDNA synthesis and real‐time qPCR

The Trizol Reagent (Invitrogen) was used to isolate the total genome RNA, the quality of which was evaluated at 260 nm by spectrophotometry. Reverse transcription was conducted using 1 µg of total RNA and M‐MLV Reverse Transcriptase Kit (Promega A3500). A 40 µl total reaction system was used in a Thermal Cycler with 96 wells (Applied Biosystems) in accordance with the following reactive processes: 72°C for 3 min, 42°C for 90 min and 70°C for 15 min. Preservation at 4°C followed. The copy number of the target gene transcription level with cDNA templates was examined by RT‐qPCR. PCR (QIAGEN) using SYBR Premix Ex TaqII (TakaraBioINC) was operated with the Rotor‐Gene Q detection system^24^ in a 20 µl setup (SYBR Premix Ex Taq II 10 µl + synthetic cDNA 1 µl + primers 0.5 µM), with the following steps: 95°C for 10 min; 95°C for 10 s, 40 cycles, 60°C for 15 s; 72°C for 20 s; and 72°C for 10 min. The value was determined, and GAPDH was chosen as the inner control.^25^ The sequences of the PCR primers (two pairs) employed in this study are listed in Table 1.

**TABLE 1:** Sequence of primers for RT‐PCR and long PCR

Target Gene	Primer Sequence	Size (bp)	Tm (°C)
Mfn1	Forward: 5′‐GGGAAGACCAAATCGACAGA‐3′	152	57
	Reverse: 5′‐CAAAACAGACAGGCGACAAA‐3′		57
Mfn2	Forward: 5′‐GAGAGGCGATTTGAGGAGTG‐3′	165	58
	Reverse: 5′‐CTCTTCCCGCATTTCAAGAC‐3′		56
Drp1	Forward: 5′‐GCCCGTGGATGATAAAAGTG‐3′	215	56
	Reverse: 5′‐TGGCGGTCAAGATGTCAATA‐3′		56
PGC‐1α	Forward: 5′‐GGACGAATACCGCAGAGAGT‐3′	201	59
	Reverse: 5′‐CCATCATCCCGCAGATTTAC‐3′		56
Nrf1	Forward: 5′‐AAACCGAACACATGGCTACC‐3′	168	58
	Reverse: 5′‐CTGCCGTGGAGTTGAGTATG‐3′		58
Tfam	Forward: 5′‐TCACCTCAAGGGAAATTGAAG‐3′	241	55
	Reverse: 5′‐CCCAATCCCAATGACAACTC‐3′		56
Long Fragment	Forward:5′‐AAAATCCCCGCAAACAATGACCACCC‐3′	13400	72
	Reverse: 5′‐GGCAATTAAGAGTGGGATGGAGCCAA‐3′		72
Shrot Fragment	Forward: 5′‐CCTCCCATTCATTATCGCCGCCCTGC‐3′	235	60
	Reverse: 5′‐GTCTGGGTCTCCTAGTAGGTCTGGGAA‐3′		60
Bax	Forward: 5′‐GCGATGAACTGGACAACAAC‐3′	200	57
	Reverse: 5′‐GATCAGCTCGGGCACTTTAG‐3′		58
Bcl‐2	Forward: 5′‐CGAGTGGGATACTGGAGATGA‐3′	236	58
	Reverse: 5′‐ GACGGTAGCGACGAGAGAAG‐3′		59
Caspase‐3	Forward: 5′‐CCCATCACAATCTCACGGTAT‐3′	195	57
	Reverse: 5′‐GGACGGAAACAGAACGAACA‐3′		58
Caspase‐9	Forward: 5′‐GCCTCTGCTTTGTCATGGAG‐3′	181	56
	Reverse: 5′‐AGCATGAGGTTCTCCAGCTT‐3′		56
PI3K	Forward: 5′‐TCACCTCCCTGATTGGCTAC‐3′	220	58
	Reverse: 5′‐CCACGATGGATGACAATGAA‐3′		55
Akt	Forward: 5′‐CGAGTCCCCACTCAACAACT‐3′	231	59
	Reverse: 5′‐GGTGAACCTGACCGGAAGTC‐3′		60
Bad	Forward: 5′‐GAGCTGACGTACAGCGTTGA‐3′	153	60
	Forward: 5′‐CCTGAGGGCTGTCCAGTAAC‐3′		60
PARP	Forward: 5′‐AAGCCTGGCACTAAGTCGAA‐3′	164	59
	Forward: 5′‐ATAGAGTAGGCGGCCTGGAT‐3′		60
Cyc‐c	Forward: 5′‐GGACAGCCCCGATTTAAGTA‐3′	121	57
	Forward: 5′‐TCAATAGGTTTGAGGCGACAC‐3′		58
GAPDH	Forward: 5′‐ AGGTCGGTGTGAACGGATTTG ‐3′	20	58
	Reverse: 5′‐ GGGGTCGTTGATGGCAACA‐3′		58

### 2.15 Target protein detection

Total proteins were drawn from myocardial tissues by using a RIPA lysis buffer. Protein concentration was measured using the BCA protein assay kit. An equal amount of total protein was dominated to sodium dodecyl sulphate‐polyacrylamide gel electrophoresis (8%–12%), and distracting the proteins to PVDF membrane. A skim milk solution was used as a blocking agent. The membranes were incubated overnight, separately, with anti‐GAPDH, anti‐PGC‐1α, anti‐TFam, anti‐Mfn1/2, anti‐Drp1, anti‐Nrf1, anti‐PI3K, anti‐p‐Akt, anti‐Akt, anti‐p‐Bad, anti‐Bad, anti‐Bcl‐2, anti‐Bax, anti‐cleaved caspase‐9/3, anti‐caspase‐9/3, anti‐Cyt‐c and anti‐PARP antibodies (Table 2). Subsequently, the membrane was incubated with secondary HRP‐conjugated goat antibodies (anti‐rabbit) (Santa Cruz Biotechnology). The target proteins (Bax [green]/Bcl‐2 [red]) in the myocardial tissues were visualized using the enhanced chemiluminescence kit (Thermo Fisher Scientific). The target proteins were then subjected to densitometry using the Alpha View software (Cell Biosciences).

**TABLE 2:** Antibodies used in the study

Antibodies	Manufacturer	Catalogue no.	Observed MW	Dilution
Anti‐PI3K	Proteintech	67071‐1‐1g	110 KDa	1:10,000
Anti‐p‐Akt	Proteintech	66444‐1‐1g	62 KDa	1:10,000
Anti‐Akt	Proteintech	10176‐2‐AP	56 KDa	1:5000
Anti‐p‐Bad	Cell signaling technology	5284S	23 KDa	1:1000
Anti‐Bad	Proteintech	10435‐1‐AP	18 KDa	1:2500
Anti‐Bcl‐2	Proteintech	26593‐1‐AP	26 KDa	1:2500
Anti‐Bax	Proteintech	50599‐2‐1g	26 KDa	1:10,000
Anti‐Caspase‐3	Proteintech	19677‐1‐AP	32 KDa	1:2000
Anti‐cleaved‐Caspase‐3	Abcam	ab49822	17 KDa	1:500
Anti‐Caspase‐9	Proteintech	10380‐1‐AP	47 KDa	1: 1000
Anti‐cleaved‐Caspase‐9	Affinity Biosciences	AF5240	10 KDa	1: 1000
Anti‐PARP1	Proteintech	13371‐1‐AP	89 KDa	1:2000
Anti‐Cyt‐c	Proteintech	12245‐1‐AP	13 KDa	1:2000
Anti‐Mfn1	Proteintech	13798‐1‐AP	86 KDa	1:1000
Anti‐Mfn2	Proteintech	12186‐1‐AP	86 KDa	1:5000
Anti‐Drp1	Proteintech	10656‐1‐AP	27 KDa	1:4000
Anti‐PGC1a	Proteintech	66369‐1‐1g	100 KDa	1:5000
Anti‐Nrf1	Proteintech	12482‐1‐AP	67 KDa	1:2500
Anti‐Tfam	Proteintech	22586‐1‐AP	25 KDa	1:5000
Anti‐GAPDH	Proteintech	60004‐1‐1g	36 KDa	1:10,000

### 2.16 Preparation and compound analysis of HQR‐containing serum

Male SD rats were intragastrically treated with normal saline (control) or HQR (9.0 g/kg/d) once daily for 7 days. Two hours after the last administration, blood was collected from the aorta ventralis, stored at 4°C for 1 h and centrifuged at 2000 rpm/min for 30 min. Serum samples from the same group were pooled, inactivated in a 56°C water bath for 30 min and sterilized by filtration. The HQR‐containing serum was stored at −80°C for subsequent vitro experiments.

The Agilent 1290UHPLC Liquid Chromatography System and Agilent MassHunter Workstation Data Acquisition Software (version B.06.00) were used for component analysis of HQR ‐containing serum. The chromatography conditions were as follows: Agilent Poroshell EC‐C18 column (internal diameter, 100 × 2.1 mm, 1.9 μm), sample size of 5 μl, temperature of 20°C, velocity flow of 0.3 ml/min, mobile phase A containing 0.1% formic acid in methanol, mobile phase B containing 0.1% formic acid in an aqueous buffer, 0–1 min mobile phase A 5%, 1–10 min mobile phase A 5–80%, 10–13 min mobile phase A 80% and stop time of 13 min. The mass spectrometry conditions were as follows: multiple reaction monitoring (MRM) and negative ion scanning mode. The internal standard was mangostin and epinastine, with an accurate weight of 1 mg standard and 5 ml of methanol diluted 500 times. Plasma samples were prepared by the methanol precipitation method.

### 2.17 Cell culture and establishment of AMI model

Rat cardiomyocytes line (H9c2) was purchased from Cell Bank of the Chinese Academy of Sciences (Shanghai, China, Item number: CBP60588). Cells (2 × 10^6^ cells) were plated in 100‐mm culture dishes and cultured in Dulbecco's modified Eagle's medium (ThermoFisher) supplemented with 10% foetal bovine serum (FBS; Gibco; Thermo Fisher Scientific, Inc.), 100 U/ml penicillin, 2 mM glutamine, 100 μg/ml streptomycin and 1 mM HEPES buffer. The cultures were incubated at 37°C in humidified air containing 5% CO_2_. The medium was replaced every other day. Palmitic acid (PA) preparation: a stock solution of sodium palmitate (5 M) was conjugated to fatty‐acid‐free bovine serum albumin (BSA) at 37°C for 1 h before addition cultured cells. The PA concentration used in this study was 0.5 mM complexed to BSA (0.2%); H9c2 cell was treated with PA (0.5 mM) for 24 h, then to build the hypoxia (5% CO_2_, 1% O_2_ and 94% N_2_) model, to mimic the myocardial hypoxia injury in vitro, and were subsequently treated with various concentrations of HQR‐containing serum (2, 4 and 8%) over 48 h.

### 2.18 Assignment of cell groups

Cells were divided into four groups as follows: control group, PA (0.5 mM)‐induced hyperlipidaemia model group, hypoxia (5% CO_2_, 1% O_2_ and 94% N_2_; 6 h, 12 h and 24 h) model group, PA + hypoxia group, PA + hypoxia + HQR‐containing serum (2, 4 and 8%) treatment group and PA + hypoxia + HQR‐containing serum + LY294002 (20 μM for 30 min) group, then used for subsequent experiments.

### 2.19 Cell viability assay

Cell viability was assessed using the Cell Counting Kit‐8 (CCK‐8). Cells (5 × 10^3^ cells/well) were plated in 96‐well plates. CCK‐8 reagent was diluted in DMEM and added to each well. Cells were incubated at 37°C for 2 h, and the absorbance was determined at 450 nm using a microplate reader. Cell viability (%) was calculated as follows: (mean absorbance in test wells)/(mean absorbance in control wells) × 100%.

### 2.20 Hoechst staining

Cells were fixed in 4% paraformaldehyde for 10 min, followed by washing in PBS and staining with Hoechst 33258 (Beyotime) for 5 min. Images were acquired under a Nikon Eclipse TE300 inverted fluorescent microscope (Tokyo, Japan) at an excitation wavelength of 350 nm and an emission wavelength of 460 nm.

### 2.21 Assessment of apoptosis with flow cytometry

Apoptosis was assessed with the Annexin V‐FITC Apoptosis Detection Kit (Annexin V‐FITC, eBioscience). Cells (1 × 10^5^ cells/ml) were seeded in 6‐well plates and incubated overnight. Cells were treated with HQR‐containing serum (2, 4 and 8%) and hypoxia (5% CO_2_, 1% O_2_ and 94% N_2_) for 24 h, followed by PA (0.5 mM) for 24 h. After washing twice with PBS, cells were suspended in binding buffer at a concentration of 1 × 10^6^ cells/ml, after which cells were incubated in 5 μl of annexin V‐FITC for 10 min and 10 μl of PI for 15 min at 4°C in the dark. Cells were analysed by flow cytometry (BD Accuril C6, BD Inc., America).

### 2.22 Electron microscopy

Cells were fixed in 4% glutaraldehyde at 4°C for approximately 2 h, washed with 0.1 M sodium dimethyl arsenate three times and centrifuged between the washing steps. The remainder of the protocol was similar to that of the in vivo experiment.

### 2.23 Preparation of mitochondrial suspension and assessment of mitochondrial function in vitro

Mitochondria were isolated using the Mitochondrial Isolation Kit (Beyotime) and maintained on ice until use. Mitochondria were resuspended in buffer to achieve a protein concentration of 5 mg/ml. Cells (1 × 10^5^ cells/ml) were plated in 6‐well plates, incubated at 37°C for 20 min and centrifuged at 600 × *g* for 3–4 min. The supernatant was discarded. Thereafter, cells were centrifuged as described. The supernatant was discarded. Lastly, cells were stained with 1× JC‐1 staining buffer and centrifuged as described. The supernatant was discarded. MMP and mPTP were visualized according the manufacturer's instructions.^19^, ^26^ All steps were carried out at 4°C.

### 2.24 Determination of protein concentrations

Cells were harvested, and proteins were extracted from cells in a lysis buffer. The protein concentration was measured using the BCA protein assay.

### 2.25 Statistical analysis

Statistical analysis was performed using SPSS 17.0 Software (SPSS Inc.). Data were expressed as the mean ± standard error. One‐way analysis of variance (ANOVA) was used to compare 4/5 independent groups. The two‐to‐two comparison among groups was used to analyse the variance The LSD‐*t* test was used to compare multiple comparisons between different groups. We regarded *p* < 0.05 represent having statistically difference.

## 3 RESULTS

### 3.1 Chemical components of HQR

The chemical components of HQR were detected by HPLC‐Q‐TOF‐MS/MS. The five chemical components were determined by strict standards, namely butane diacid (CAS: 110‐15‐6), paeoniflorin (CAS: 23180‐57‐6), ferulic acid (CAS: 537‐98‐4), astragaloside Ⅱ (CAS: 91739‐01‐4) and astragaloside Ⅰ (CAS: 84680‐75‐1), and the contents were 9.6, 50, 25, 8.5 and 4.1 mg/g, respectively. The extracted‐ion chromatograms are shown in Figure 1A, and the ESI‐Q‐TOF MS/MS spectra of the five components are shown in Figure 1B.

### 3.2 HQR improved blood fat, myocardial enzymes, cardiac function and inflammatory factors after AMI

**FIGURE 2:**
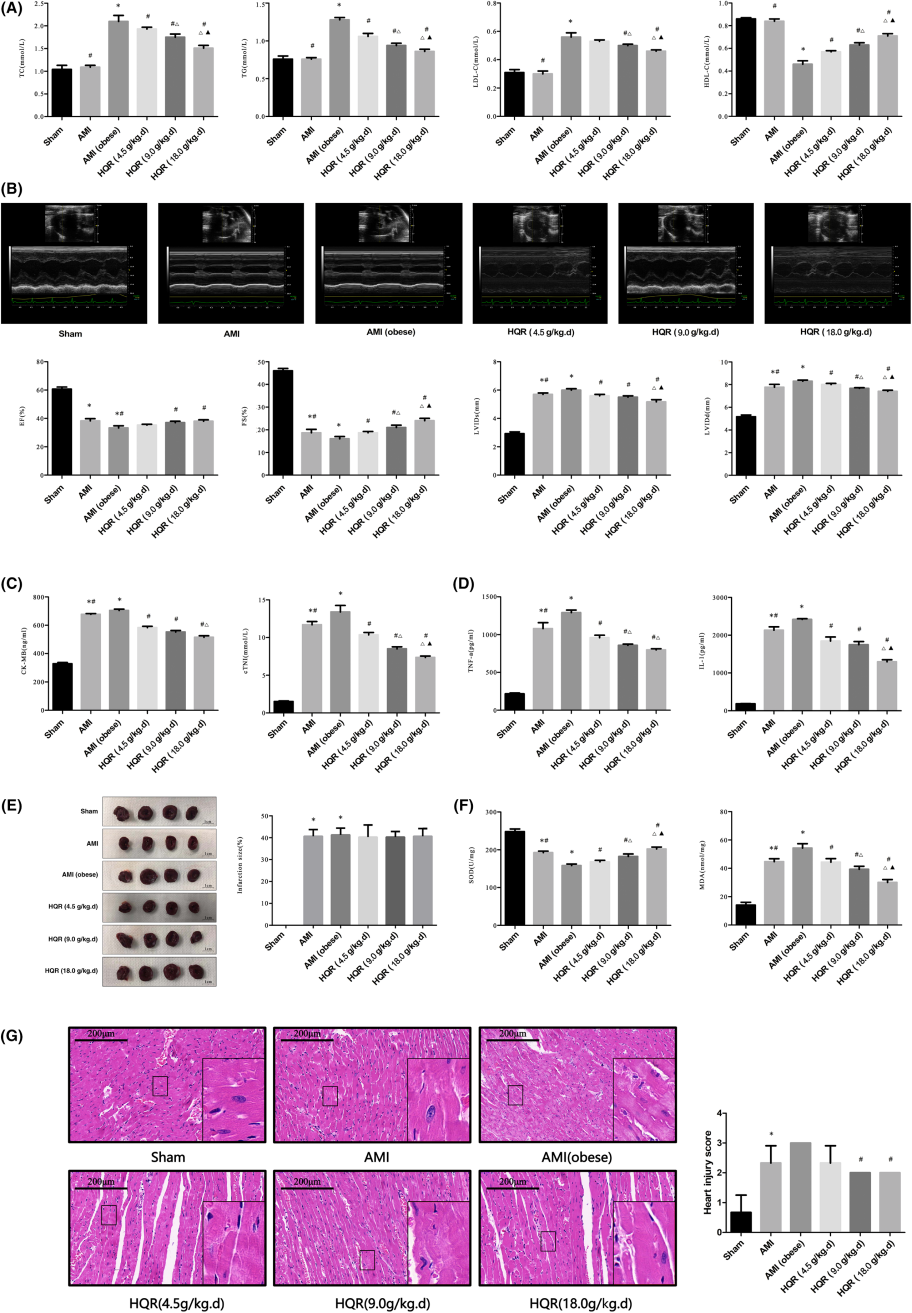
Huayu Qutan recipe (HQR) improved the blood fat, cardiac function, myocardial enzymes, inflammatory factor, imbalance between oxidation and antioxidation, and myocardial architecture after acute myocardial infarction (AMI). Rats were pretreated with HQR followed by AMI. Sham rats were used as control. Blood fat (A), cardiac function (B), myocardial enzymes (C), inflammatory factor (D), the infarction size (E), oxidation/antioxidation (F) and myocardial architecture (G) were evaluated under different groups; the scale bars represent a length of 50 μm on histology. Data are shown as mean ± SD. *p < 0.05 versus sham group, ^#^ *p* < 0.05 versus AMI (obese) group, ^Δ^ *p* < 0.05 versus HQR (4.5 g/kg.d) group, ^▲^ *p* < 0.05 versus HQR (9.0 g/kg.d) group. (*n* = 3)

Blood fat composition is shown in Figure 2A, cardiac function in Figure 2B, myocardial enzymes in Figure 2C, inflammatory factors in Figure 2D and infarction size in Figure 2E to further evaluate the standard of myocardial injury and the protective effect and mechanism of HQR.

The TC, LDL‐C and TG increased in the AMI (obese) group relative to that in the AMI and sham groups (*p* < 0.05). Their values decreased via pretreatment with HQR. HDL‐C decreased in the AMI (obese) group relative to the HDL‐C levels in the AMI and sham groups (*p* < 0.05), which increased via pretreatment with HQR (Figure 2A). The EF and FS decreased in the AMI and AMI (obese) groups (particularly with a large increase in the AMI (obese) group), relative to those in the sham group (*p* < 0.05). The LVIDs and LVIDd values increased in the AMI and AMI (obese) groups (markedly increased in the AMI (obese) group) relative to those in the sham group (*p* < 0.05). The values increased via pretreatment with HQR (Figure 2B). The cTNI and CK‐MB values increased in the AMI and AMI (obese) groups (particularly with a large increase in the AMI (obese) group), relative to those in the sham group (*p* < 0.05), which were decreased via pretreatment with HQR (Figure 2C). The IL‐1β and TNF‐α values increased in the AMI and AMI (obese) groups (markedly increased in the AMI (obese) group), relative to those in the sham group (*p* < 0.05), which were decreased via pretreatment with HQR (Figure 2D). The infarction size (%) increased in the AMI and AMI (obese) groups, relative to that in the sham group (*p* < 0.05), which was not decreased via pretreatment with HQR (Figure 2E).

### 3.3 HQR improved the imbalance between oxidation (MDA) and antioxidation (SOD) after AMI

The MDA levels in the AMI and AMI (obese) groups were higher than those in the sham group (*p* < 0.05). However, the high MDA level could be decreased via pretreatment with HQR. The SOD levels in the AMI and AMI (obese) groups were lower than the SOD level in the sham group (*p* < 0.05). However, pretreatment with HQR could increase SOD. (Figure 2F).

### 3.4 HQR improved the pathological structure of the myocardium after AMI

We evaluated the myocardial protective effect of HQR after AMI by HE staining of myocardial tissues (Figure 2G). Considerably disorganized myocardial cells (interstitial oedema) were observed in the AMI and AMI (obese) groups, which was remedied via pretreatment with HQR, as indicated by the alleviation of both interstitial oedema and disorganized myocardial cells. The total damage was evaluated using a histological score (Figure 2G). Statistical results indicated that the myocardial tissue damage in the AMI and AMI (obese) groups increased relative to that in the sham group (*p* < 0.05); the myocardial tissue damage was higher in the AMI (obese) group than in the AMI group (*p* < 0.05). All myocardial tissue damage scores decreased in the HQR groups (*p* < 0.05) (Figure 2G).

### 3.5 HQR relieves cell apoptosis after AMI

**FIGURE 3:**
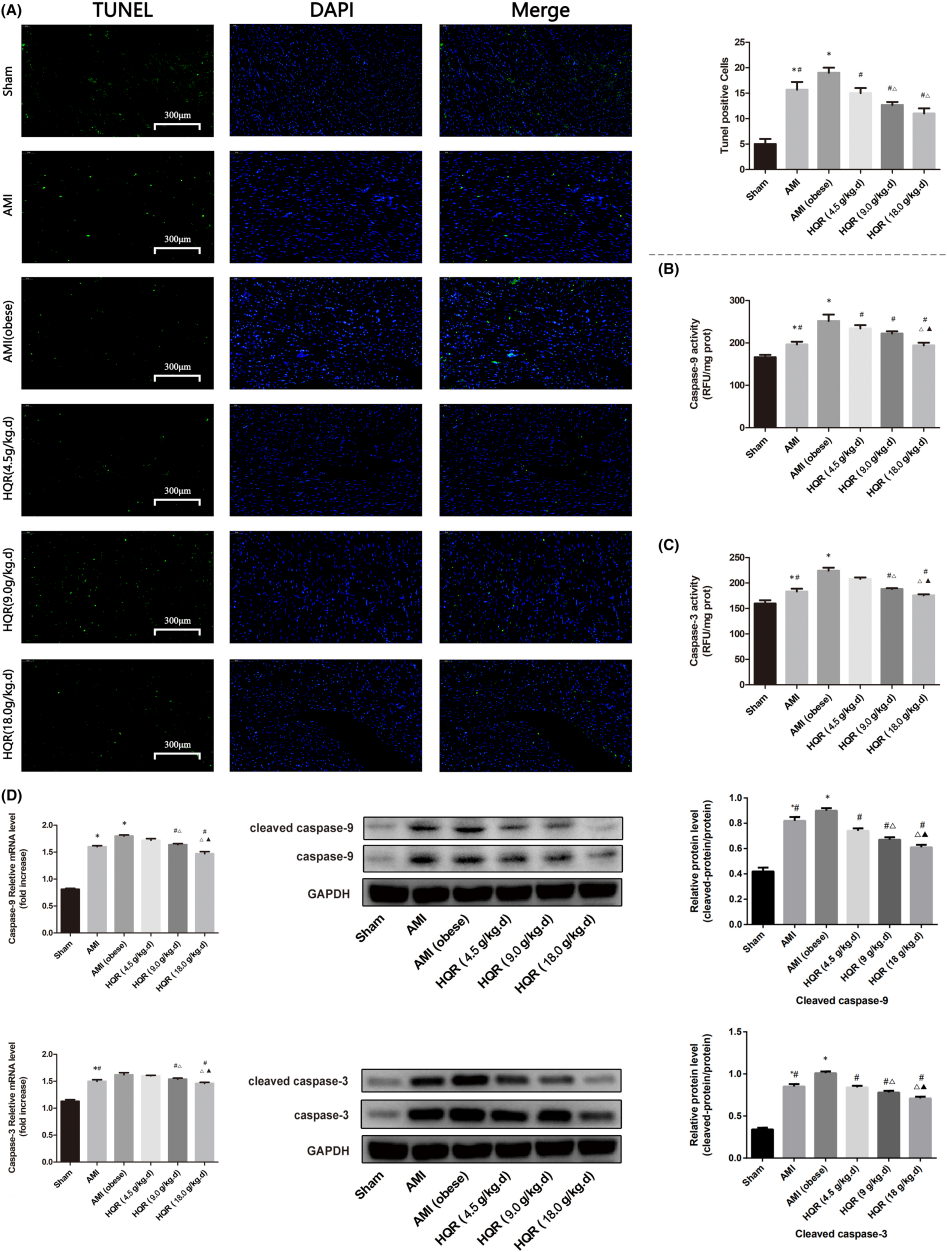
Huayu Qutan recipe (HQR) inhibited myocardial cells apoptosis after acute myocardial infarction (AMI). Rats were pretreated with HQR followed by AMI. Sham rats were used as control. Sham rats were used as control. Representative apoptosis of myocardial cells, TUNEL‐positive cells, the scale bars represents a length of 300 μm on histology (A), the activity of caspase‐9/3 (B, C) and the mRNA levels of caspase‐9/3, and protein levels of cleaved caspase‐9/3 (D) were evaluated under different groups. Data are shown as mean ± SD. *p < 0.05 versus sham group, ^#^ *p* < 0.05 versus AMI (obese) group, ^Δ^ *p* < 0.05 versus HQR (4.5 g/kg.d) group, ^▲^ *p* < 0.05 versus HQR (9.0 g/kg.d) group. (*n* = 3)

**FIGURE 4:**
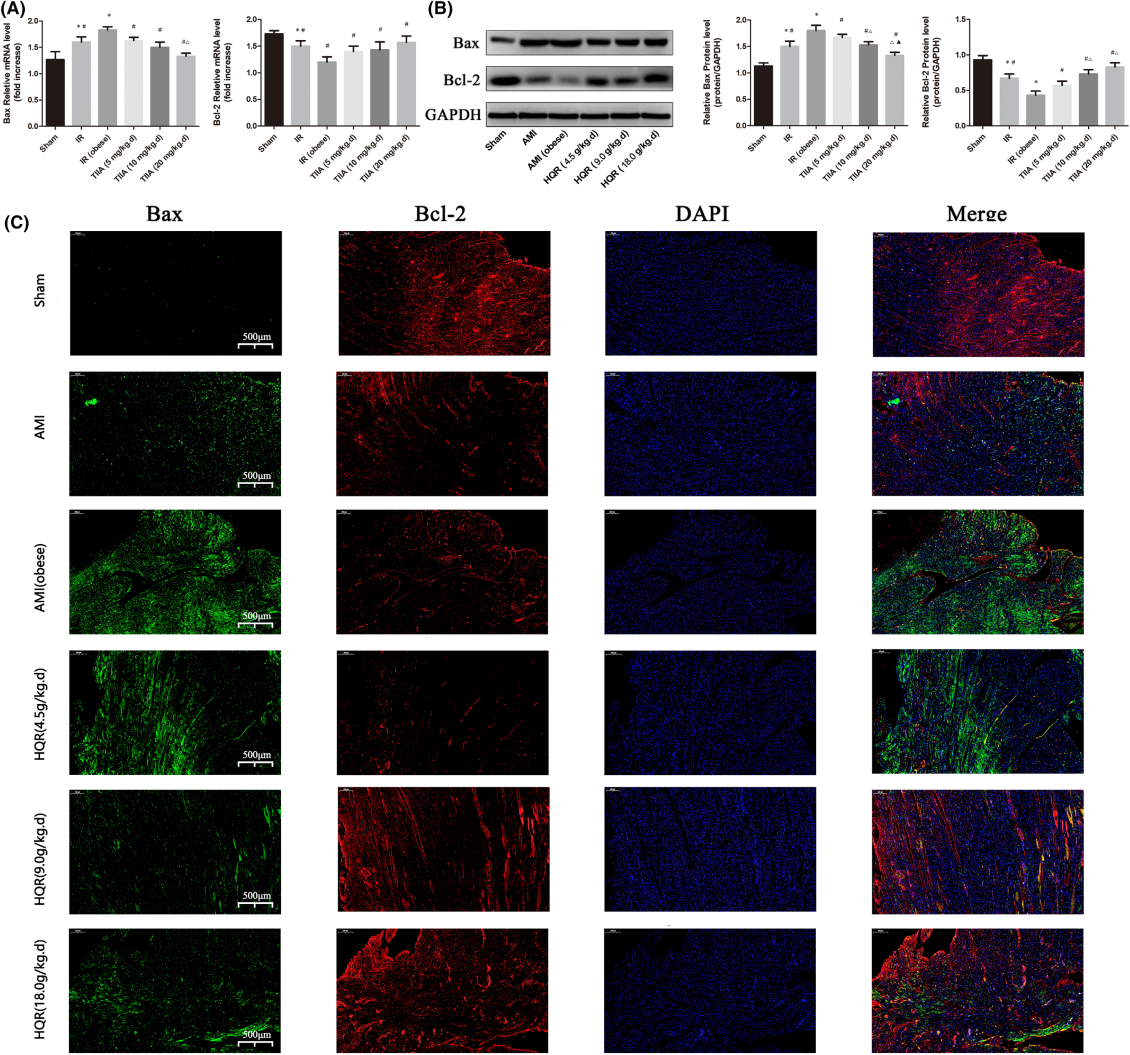
Huayu Qutan recipe (HQR) decreased Bax and increased Bcl‐2 (Immunofluorescence results). The expression of Bax and Bcl‐2 in mRNA (A) and protein (B) level. Data are shown as mean ± SD. *p < 0.05 versus sham group, ^#^ *p* < 0.05 versus AMI (obese) group, ^Δ^ *p* < 0.05 versus HQR (4.5 g/kg.d) group, ^▲^ *p* < 0.05 versus HQR (9.0 g/kg.d) group. (*n* = 3). Immunofluorescence results of Bax (green) and Bcl‐2 (red) expression in nephridial tissue (C), the scale bars represent a length of 500 μm on histology

The TUNEL assay was used to determine the protective effect of HQR on myocardial tissue cell apoptosis after AMI in rats with obesity (Figure 4A). The number of apoptotic cells in myocardial tissues in the AMI and AMI (obese) groups increased relative to that in the sham group (*p* < 0.05). However, pretreatment with HQR could reduce the number of myocardial tissues apoptotic cells (Figure 3A). The caspase‐9/3 activity in myocardial cells in the AMI and AMI (obese) groups was higher than that in the sham group (*p* < 0.05). However, pretreatment with HQR decreased the caspase‐9/3 activity of the myocardial cells (Figure 3B,C). Western blot analysis was performed to assess the protein levels of cleaved caspase‐9/3 (Figure 3D). The protein levels of cleaved caspase‐9/3 in the AMI and AMI (obese) groups increased relative to that in the sham group (*p* < 0.05). However, pretreatment with HQR reduced the mRNA levels of caspase‐9/3 and protein levels of cleaved caspase‐9/3 (*p* < 0.05) (Figure 3D).

### 3.6 HQR improved expression of apoptosis‐related genes (Bax/Bcl‐2) after AMI

The mRNA and protein expression of Bax was increased in the AMI group (markedly increased in the AMI [obese] group), relative to that in the sham group (*p* < 0.05). The mRNA and protein expression of Bax decreased after pretreatment with HQR (*p* < 0.05). The mRNA and protein expression of Bcl‐2 was decreased in the AMI group (markedly reduced in the AMI [obese] group), relative to that in the sham group (*p* < 0.05). (Figure 4A,B). The immunofluorescence results for Bax/Bcl‐2 expression in the myocardial tissues showed that Bax expression increased, whereas Bcl‐2 expression decreased in the AMI group (markedly reduced in the AMI [obese] group). Bax expression decreased, whereas Bcl‐2 increased after pretreatment with HQR (Figure 4C).

### 3.7 HQR improves mitochondrial function after AMI

**FIGURE 5:**
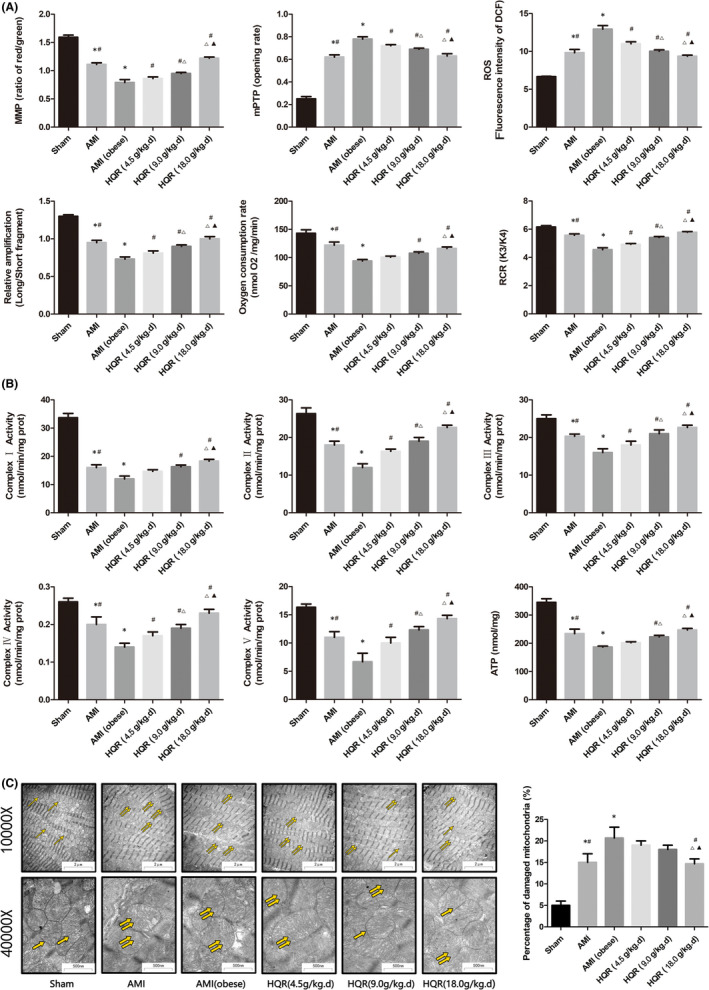
Huayu Qutan recipe (HQR) preserved myocardial mitochondrial function in acute myocardial infarction (AMI)‐induced myocardial injury. The MMP (ratio of red/green), the opening of mPTP (%), the mitochondrial ROS, the mtDNA damage (ratio of long/short fragments), the mitochondrial RCR, mitochondrial oxygen consumption rate (A), the mitochondrial respiratory chain complex enzymes (Ⅰ, Ⅱ, Ⅲ, Ⅳ and Ⅴ) and ATP (B) were recorded above. Electron microscope pictures (10,000×, 40,000×) of rats myocardial tissue after AMI, the scale bars represent a length of 2 μm and 500 nm on tissues, respectively. Abnormal mitochondrial (paired yellow arrow) morphology show that mitochondrial membrane rupture or swellings, normal mitochondrial (single yellow arrow) morphology type show that mitochondrial membrane smooth and inner carinulae distinct (C), and percentage of damaged mitochondria (C). Data are shown as mean ± SD. *p < 0.05 versus sham group, ^#^ *p* < 0.05 versus AMI (obese) group, ^Δ^ *p* < 0.05 versus HQR (4.5 g/kg.d) group, ^▲^ *p* < 0.05 versus HQR (9.0 g/kg.d) group. (*n* = 3)

We examined the MMP (red/green ratio), the mPTP opening (%), ROS, mtDNA, oxygen consumption rate, RCR, the activity of the mitochondrial respiratory chain complex enzymes (Ⅱ, Ⅲ, Ⅳ and Ⅴ), and ATP to further evaluate the mitochondrial function of myocardial tissues after AMI and protective effect of HQR. The level of ROS and mPAP opening (%) can be increased after AMI; obviously, the obese combined with AMI (*p* < 0.05). The level of ROS and mPAP opening (%) was reduced by pretreatment with HQR (*p* < 0.05). AMI reduced the mitochondrial RCR, oxygen consumption rate and the MMP (red/green ratio), and obviously, the obese combined with AMI (*p* < 0.05). These indices could be increased by pretreatment with HQR (*p* < 0.05). Real‐time qPCR can determine the level of damage to mtDNA. The long fragments‐to‐short fragments ratio decreased after AMI (markedly decreased in the AMI [obese] group (*p* < 0.05)). Pretreatment with HQR could increase the long/short mtDNA fragment ratio (*p* < 0.05) (Figure 5A). AMI reduced the activity of the mitochondrial respiratory chain complex enzymes (1, Ⅱ, Ⅲ, Ⅳ and Ⅴ) and ATP (largely decreased in the AMI [obese] group (*p* < 0.05)). Pretreatment with HQR could increase enzymatic activities and ATP levels (*p* < 0.05) (Figure 5B).

### 3.8 Morphological changes in myocardial mitochondria after AMI

Using an electron microscope, we evaluated the morphological changes in myocardial mitochondria to determine the myocardial damage and the protective effect of HQR. (Figure 3B).

Electron microscopy images (10,000× and 40,000×) revealed abnormal morphological mitochondria in the cells of the myocardial tissues in the AMI and AMI (obese) groups. The membrane appeared swollen and ruptured (denoted by paired yellow arrows) after AMI. Meanwhile, the sham group revealed normal morphological mitochondria (denoted by a single yellow arrow). The number of abnormal morphological mitochondria increased in the AMI and AMI (obese) groups (markedly increased in the AMI [obese] group) than in the sham group (*p* < 0.05). Pretreatment with HQR decreased the number of abnormal morphological mitochondria (*p* < 0.05) (Figure 5C).

### 3.9 HQR improved mitochondrial dynamics and biogenesis after AMI

**FIGURE 6:**
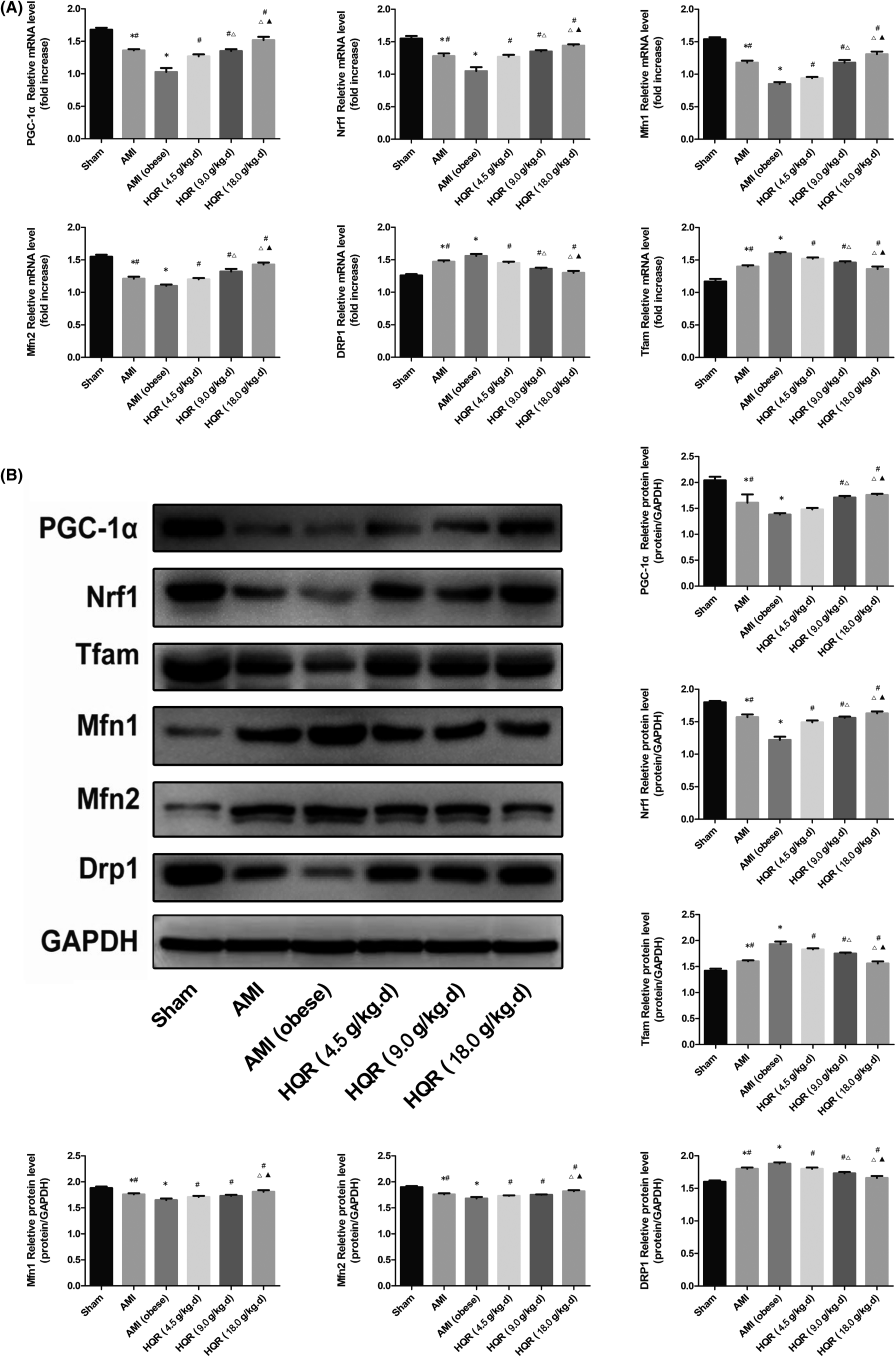
Huayu Qutan recipe (HQR) preserved mitochondrial biogenesis and dynamics in acute myocardial infarction (AMI)‐induced myocardial injury. The expression of PGC‐1α, Nrf1 and Tfam in mRNA (A) and protein (B) level. The expression of Mfn1, Mfn2 and Drp1 in mRNA and protein level. Data are shown as mean ± SD. *p < 0.05 versus sham group, ^#^ *p* < 0.05 versus AMI (obese) group, ^Δ^ *p* < 0.05 versus HQR (4.5 g/kg.d) group, ^▲^ *p* < 0.05 versus HQR (9.0 g/kg.d) group. (*n* = 3)

Using real‐time qPCR and Western blot analysis to determine the gene expression of mitochondrial dynamics and biogenesis in mRNA and protein levels. Nrf1, PGC‐1α and Tfam served as mitochondrial biogenesis indices, Drp1 as a mitochondrial fission index and mitofusins (Mfn1/2) as mitochondrial fusion indices. The results of this study demonstrated that the mRNA and protein levels of Nrf1, PGC‐1α, Tfam and Drp1 could be reduced in the AMI and AMI (obese) groups (with a large reduction in the AMI [obese] group) relative to those in the sham group (*p* < 0.05). Pretreatment with HQR could increase the mRNA and protein expression of those genes (*p* < 0.05). The mRNA and protein levels of Mfn1/2 increased in the AMI and AMI (obese) groups (markedly increased in the AMI [obese] group), compared with sham (*p* < 0.05). Pretreatment with HQR could decrease the expression of those genes in mRNA and protein (*p* < 0.05) (Figure 6).

### 3.10 HQR modulates the PI3K/Akt/Bad pathway *in vivo*

**FIGURE 7:**
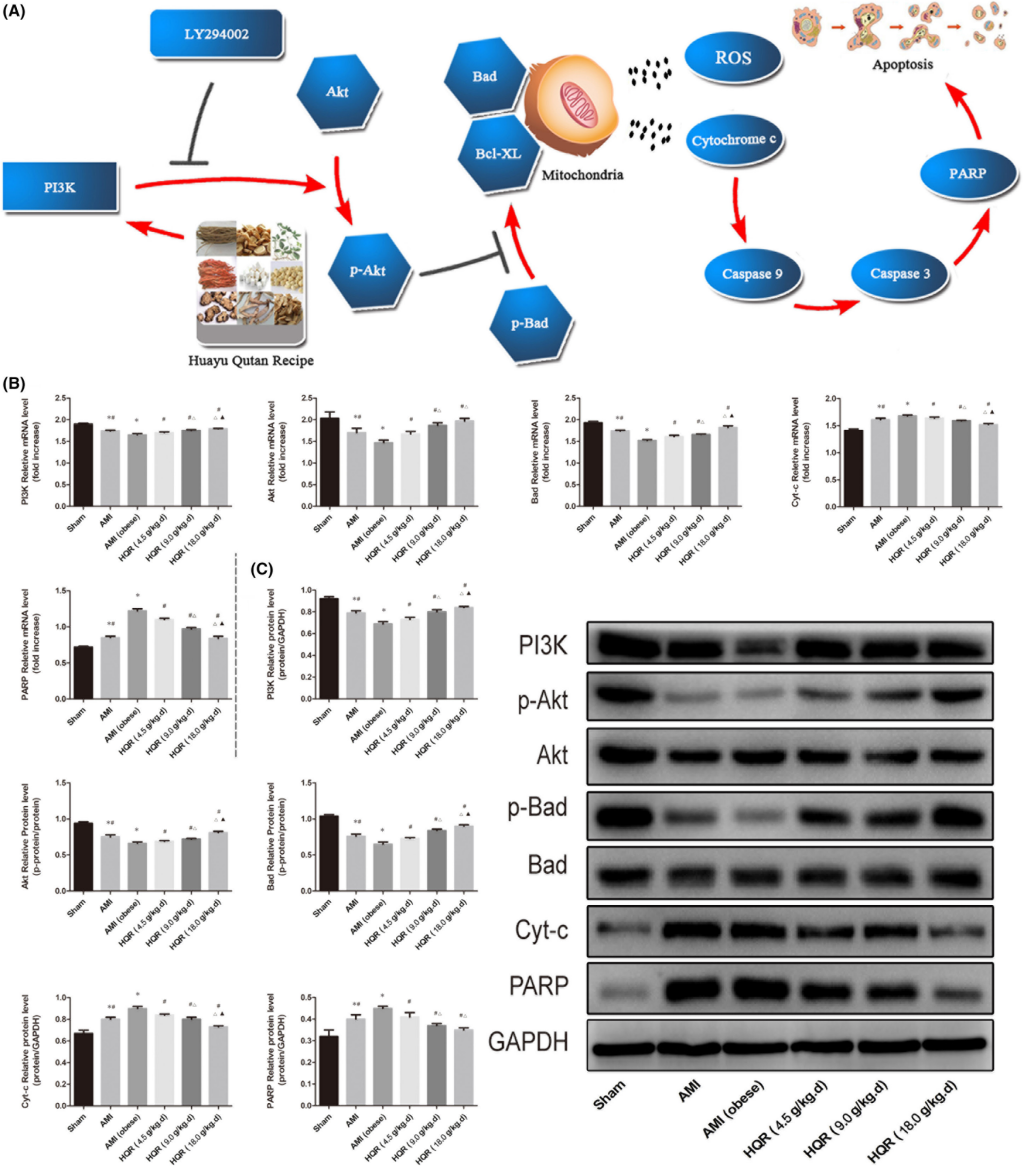
Huayu Qutan recipe (HQR) modulated myocardial PI3K/Akt/Bad pathway. (A) Myocardial mitochondrial dysfunction occurs following AMI and can lead to myocardial apoptosis, which can be aggravated by obesity. HQR can relieve myocardial apoptosis by improving mitochondrial function via the PI3K/Akt/Bad pathway in rats with obesity. We suggested that HQR might be used as a potential therapeutic agent for injury following AMI, and mitochondria can be seen as a potential therapeutic target; the expression of PI3K, p‐Akt, Akt, p‐Bad, Bad, Cyt‐c and PARP in mRNA (B) and protein (C) level. Data are shown as mean ± SD. *p < 0.05 versus sham group, ^#^ *p* < 0.05 versus AMI (obese) group, ^Δ^ *p* < 0.05 versus HQR (4.5 g/kg.d) group, ^▲^ *p* < 0.05 versus HQR (9.0 g/kg.d) group. (*n* = 3)

We used real‐time qPCR and Western blot analysis to determine the target gene expression via the PI3K/Akt/Bad pathway in mRNA and protein. The mRNA expression of caspase‐9/3, Cyt‐c and PARP increased in the AMI group (particularly in the AMI [obese] group), relative to the sham group (*p* < 0.05). The mRNA expression of these indices decreased after pretreatment with HQR (*p* < 0.05). The mRNA expression of PI3K, Bad and Akt decreased in the AMI group (markedly reduced in the AMI [obese] group), relative to that in the sham group (*p* < 0.05). The mRNA expression of these genes could be increased via pretreatment with HQR (*p* < 0.05) (Figure 7A). Western blot analysis of target genes in the PI3K/Akt/Bad pathway showed consistent results in protein levels (Figure 7B). HQR promoted Bad and Akt phosphorylation (i.e. increased the p‐Bad/Bad and p‐Akt/Akt ratios) (Figure 7B).

### 3.11 HQR alleviated hypoxia‐induced cell death and increased cell apoptosis in vitro

**FIGURE 8:**
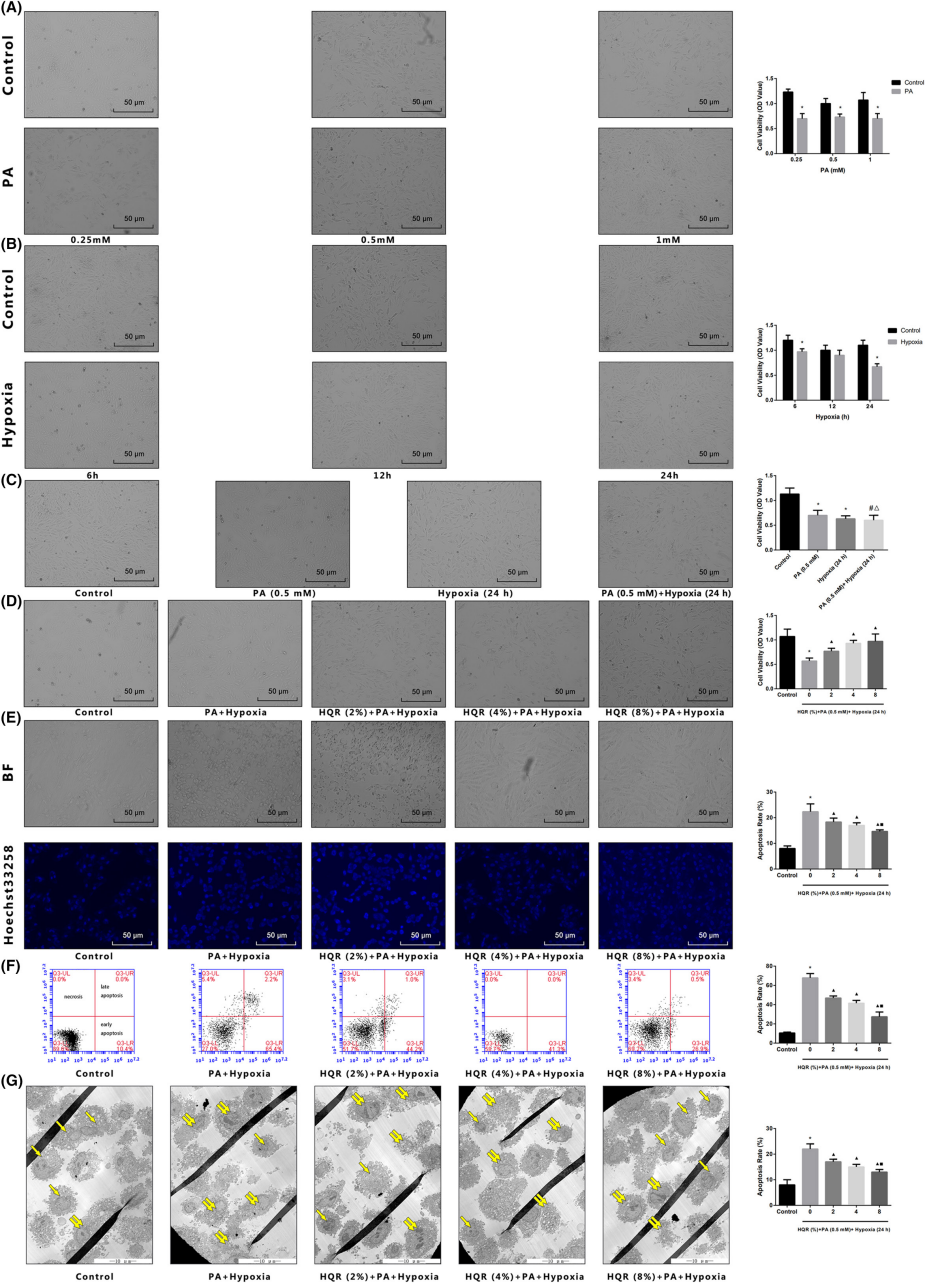
Huayu Qutan recipe (HQR) improved decreased cell viability, increased cell apoptosis caused by hypoxia in vitro. HQR‐containing serum inhibited loss of cells viability induced by hypoxia. (A) Cells were treated with PA (0.25 mM, 0.5 mM and 1 mM); (B) cells were treated with hypoxia (6 h, 12 h and 24 h); (C) cells were treated with PA (0.5 mM) + hypoxia (24 h); (D) cells were treated with HQR‐containing serum (2, 4 and 8%) then treated with PA (0.5 mM) + hypoxia (24 h); (E) Hoechst 33258 staining was used to detect the apoptosis and counted the percentage of apoptotic cells (with a dim fluorescent signal indicating of viability and a bright fluorescent signal indicating apoptosis); (F) cell apoptosis was measured by flow cytometry and counted the percentage of apoptotic cells: provided 2‐dimensional graphical representations of PI/annexin V‐FITC plots. ‘Early apoptosis’ was defined as cells positive for annexin V‐FITC only. ‘Late apoptosis’ was defined as cells positive for annexin V‐FITC and PI. ‘Necrosis’ was defined as cells positive for PI only; (G) cell apoptosis was observed by electron microscope pictures (2500×); the scale bars represent a length of 10 μm on cells, respectively, apoptotic cell (paired yellow arrow) and normal cell (single yellow arrow). Data are shown as mean ± SD. *p < 0.05 versus control group, ^#^ *p* < 0.05 versus PA group, ^Δ^ *p* < 0.05 versus hypoxia group, ^▲^ *p* < 0.05 versus PA + hypoxia group, ^□^ *p* < 0.05 versus PA + hypoxia + HQR (2%) group, ^■^ *p* < 0.05 versus PA + hypoxia + HQR (4%) group. (*n* = 3)

To evaluate the effects of HQR‐containing serum and hypoxia on cell function, a cell viability assay was used. Compared to the control group, cell viability decreased by treatment with PA (0.25 mM, 0.5 mM and 1 mM), hypoxia (6 h, 12 h and 24 h) and PA + hypoxia (*p* < 0.05). The results of light microscopy revealed normal cells with long fusiform shapes in the control group. However, PA, hypoxia and PA + hypoxia treatment altered cell shape by shrinking cells and disrupting cell–cell interactions. Cell viability was not affected by HQR‐containing serum (2, 4 and 8%) but it was affected by pretreatment with PA, hypoxia and PA + hypoxia followed by treatment with HQR‐containing serum. HQR‐containing serum attenuated the decreased cell viability dose‐dependently (*p* < 0.05) (Figure 8A–D). At the highest concentration, HQR‐containing serum (8%) attenuated the decreased cell viability dose‐dependently (*p* < 0.05) (Figure 8D). As such, 0.5 mM PA and 24 h hypoxia were selected for subsequent experiments.

To assess apoptosis, cells were stained with Hoechst 33258, with a dim fluorescent signal indicating of viability and a bright fluorescent signal indicating apoptosis. Compared to the control group, PA, hypoxia and PA + hypoxia induced apoptosis. At the highest concentration, HQR‐containing serum (8%) attenuated the decreased apoptosis dose‐dependently (*p* < 0.05) (Figure 8E).

To further evaluate anti‐apoptotic effects of HQR‐containing serum, we assessed apoptosis by flow cytometry. Compared with the control group, the apoptotic (late and early) rate increased in the hypoxia and PA + hypoxia groups (*p* < 0.05). The apoptotic (late and early) rate was lower in cells pretreated with HQR‐containing serum at concentrations of 2, 4 and 8% than that in untreated cells (*p* < 0.05), and the effects were dose‐dependent (*p* < 0.05) (Figure 8E). The results of electron microscopy revealed that untreated cells were healthy, whereas hypoxia and PA + hypoxia caused apoptosis, and HQR‐containing serum (2, 4 and 8%) can inhibit apoptosis induced by hypoxia and PA + hypoxia (Figure 8F).

### 3.12 HQR improved PA + hypoxia‐induced mitochondrial dysfunction in vitro

**FIGURE 9:**
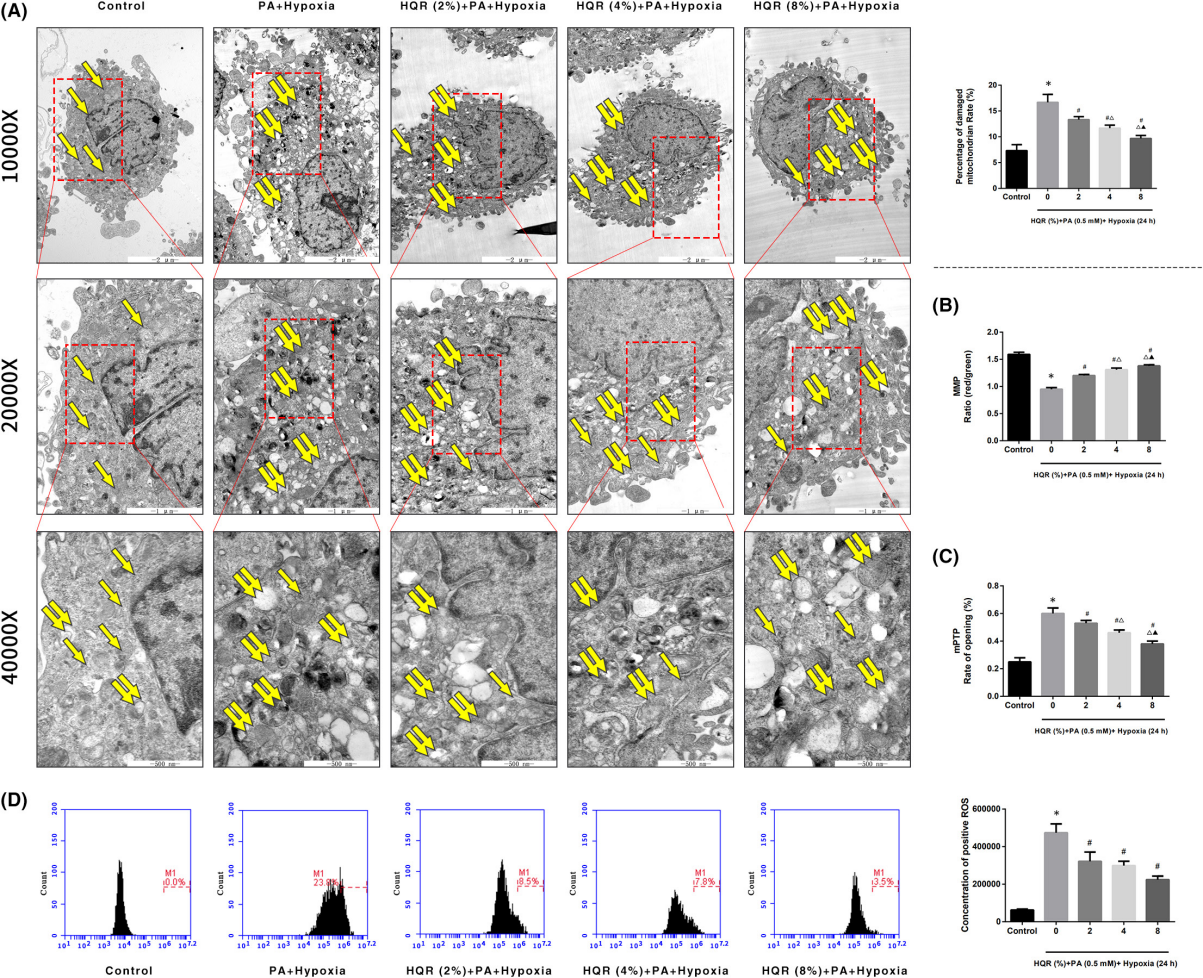
Huayu Qutan recipe (HQR) improved mitochondrial dysfunction caused by hypoxia in vitro. Cells were treated with PA and hypoxia, then treated with HQR‐containing serum (2, 4, 8%). (A) Electron microscope pictures (10,000×; 20,000×; 40,000×) of cells, the scale bars represent a length of 2 μm, 1 μm and 500 nm on cells, respectively. Abnormal mitochondrial (paired yellow arrow) morphology show that mitochondrial membrane rupture or swellings, normal mitochondrial (single yellow arrow) morphology type show that mitochondrial membrane smooth and inner carinulae distinct and percentage of damaged mitochondria; (B) the MMP (ratio of red/green); (C) the opening of the mPTP (%); (D) the ROS levels were measured by flow cytometry. Data are shown as mean ± SD. *p < 0.05 versus control group, ^#^ *p* < 0.05 versus PA (0.5 mM) + Hypoxia (24 h) group, ^Δ^ *p* < 0.05 versus HQR (2%) + PA (0.5 mM) + Hypoxia (24 h) group, ^▲^ *p* < 0.05 versus HQR (4%) + PA (0.5 mM) + Hypoxia (24 h) group. (*n* = 3)

To further assess the protective effects of HQR‐containing serum, mitochondrial function was assessed by observing changes in mitochondrial morphology, the MMP and the mPTP (%). Compared to cells of the control group, which had normal mitochondria (single yellow arrow), the results of electron microscopy revealed the presence of abnormal mitochondria, with membrane rupturing or swelling (paired yellow arrows), in cells of the hypoxia and PA + hypoxia groups. The percentage of damaged mitochondria was higher in the hypoxia and PA + hypoxia groups than that in the control group (*p* < 0.05). The percentage of damaged mitochondria decreased after treatment with HQR‐containing serum at concentrations of 2, 4 and 8% (*p* < 0.05) (Figure 9A).

Compared to the control group, the MMP decreased in the hypoxia and PA + hypoxia groups (*p* < 0.05). Cells pretreated with 2, 4 and 8% HQR‐containing serum had an increased MMP (*p* < 0.05) (Figure 9B). Compared to the control group, the mPTP (%) increased in the CTX group (*p* < 0.05). Cells pretreated with 2, 4 and 8% HQR‐containing serum had a decreased mPTP (%) (*p* < 0.05) (Figure 9C). Compared to the control group, the ROS levels increased in the hypoxia and PA + hypoxia groups (*p* < 0.05), cells pretreated with 2, 4 and 8% HQR‐containing had an decreased ROS levels (*p* < 0.05) (Figure 9D). As such, 8% HQR‐containing serum was selected for subsequent experiments.

### 3.13 HQR modulates the PI3K/Akt/Bad pathway to inhibit apoptosis and mitochondrial dysfunction in vitro

**FIGURE 10:**
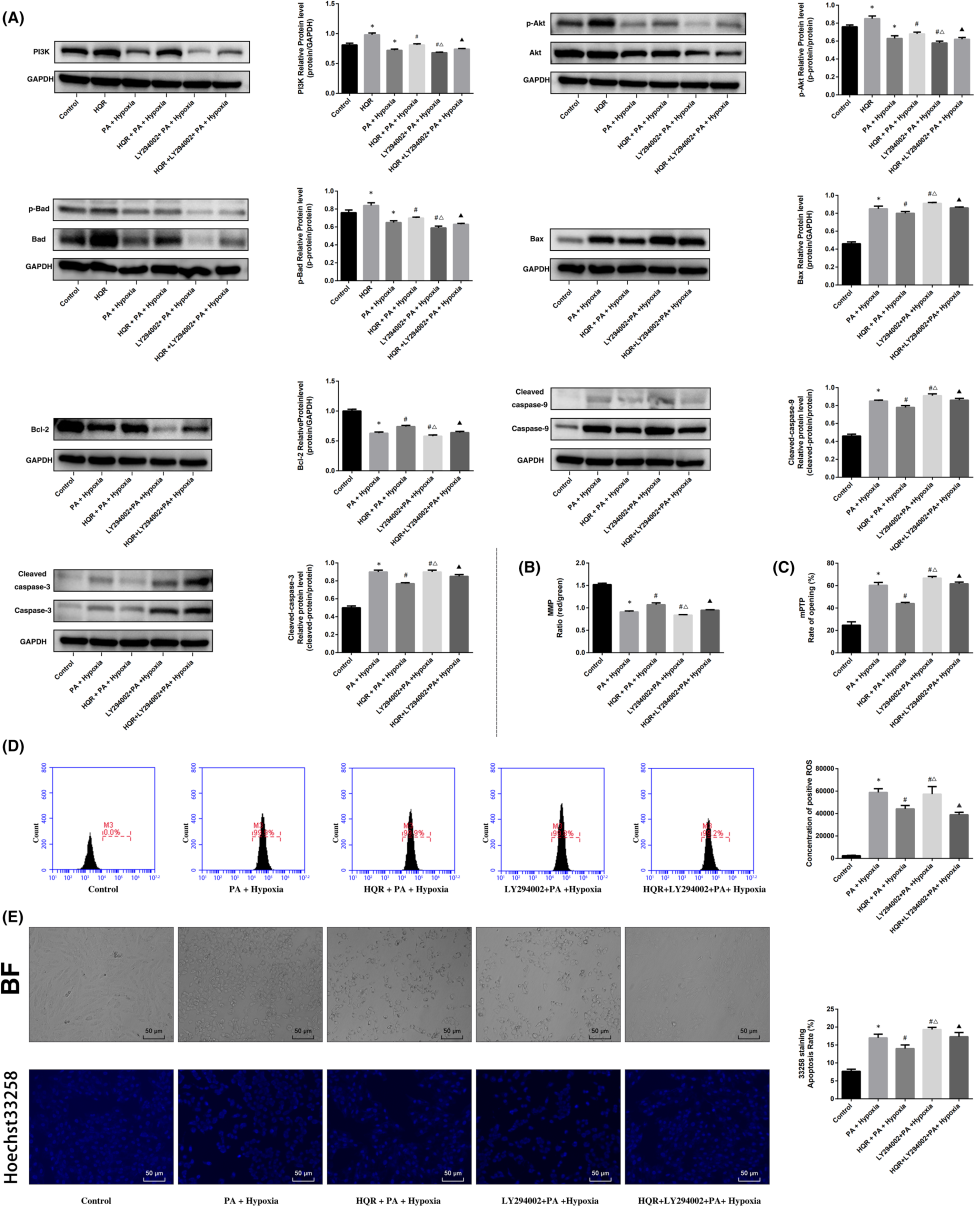
Huayu Qutan recipe (HQR) activated PI3K/Akt/Bad pathway to inhibit apoptosis and mitochondrial dysfunction in vitro. To investigate whether the anti‐apoptotic effect of HQR‐containing serum (8%) was associated with the PI3K/Akt/Bad pathway. LY294002 (20 μM) was the inhibitor of PI3K. (A) We used Western blot to detect the target genes of the PI3K/Akt/Bad pathway in protein level; (B) the MMP (ratio of red/green); (C) the opening of mPTP (%); (D) the ROS levels were measured by flow cytometry; and (E) Hoechst 33258 staining was used to detect the apoptosis and counted the percentage of apoptotic cells. Data are shown as mean ± SD. *p < 0.05 versus control group, ^#^ *p* < 0.05 versus PA + Hypoxia group, ^Δ^ *p* < 0.05 versus HQR + PA + Hypoxia group, ^▲^ *p* < 0.05 versus LY294002 + PA + Hypoxia group. (*n* = 3)

To investigate whether the anti‐apoptotic effects of HQR‐containing serum were associated with the PI3K/Akt/Bad pathway, LY294002 (PI3K inhibitor) was used. Compared to the control group, hypoxia and PA + hypoxia decreased Akt and Bad phosphorylation and the expression of PI3K (*p* < 0.05), whereas HQR increased the Akt and Bad phosphorylation and the expression of PI3K (*p* < 0.05), but the LY294002 can decreased the Akt and Bad phosphorylation and the expression of PI3K (*p* < 0.05). The protein expression of caspase‐9/3, Cyt‐c and Bax increased in the hypoxia and PA + hypoxia groups, relative to the control group (*p* < 0.05). The protein expression of these indices decreased after pretreatment with HQR (*p* < 0.05), but the LY294002 can reverse the effects of HQR. (Figure 10A). Compared to the control group, the MMP level (*p* < 0.05) was decreased but the mPTP level (*p* < 0.05) was increased in the hypoxia and PA + hypoxia groups. Compared with the hypoxia and PA + hypoxia groups, the MMP level (*p* < 0.05) was increased but the mPTP level (*p* < 0.05) was decreased after HQR and LY294002 treatment can reverse the effects of HQR (Figure 10B,C). Compared to the control group, the ROS levels increased in the hypoxia and PA + hypoxia groups (*p* < 0.05), cells pretreated with HQR can decrease ROS levels and LY294002 treatment can reverse the effects of HQR (*p* < 0.05) (Figure 10D).

## 4 DISCUSSION

As the leading cause of death worldwide, ischaemic heart disease (IHD) leads to more than 9 million deaths annually. AMI is a manifestation of IHD in which coronary atheromatous plaque ruptures, inducing an acute thrombotic occlusion of the coronary artery and seriously confining or completely obstructing blood flow to myocardial tissues, depriving myocardial cells (namely AMI) of nutrients and oxygen, leading to their death.^9^ Hyperlipidaemia can also aggravate myocardial damage.^27^ Among the postulated mechanisms for the occurrence and development of AMI, mitochondria have drawn considerable attention.^28^, ^29^, ^30^

As the energy powerhouse of the cell, mitochondria produce the largest amount of ATP.^31^ After an injury, the mitochondrial membrane potential is reduced. Harmful by‐products of ATP, such as pro‐apoptotic factors (Cyt‐c) and ROS, are discharged. Cyt‐c can exert harmful effects on the function and fate of the cell.^32^ Mitochondrial dysfunction can be viewed as a predictive factor for cell death.^33^ In the current study, we chose the non‐infarcted myocardial tissue because improving and reducing the severity of the injury of non‐infarcted myocardial tissue is crucial during clinical treatment.

The major findings in the current study are that myocardial injury is more severe after AMI combined with obesity and that HQR exerts potentially protective effects on myocardial injury induced by AMI. This study also determines and mechanisms of HQR. These findings can be verified by *in vivo* models. Moreover, the mitochondrial function was lower in rats with obesity than those without obesity. This occurrence is even more pronounced after AMI. Post‐AMI rats with obesity exhibited more severe damage and apoptosis in myocardial cells, compared with other rats. To the best of our knowledge, this study is the first to examine the combined effects of AMI and obesity on mitochondrial function and apoptosis in myocardial cells. The key to the preventive treatment may be the improvement of hyperlipidaemia. In accordance with the principle of TCM, the pathogenesis of hyperlipidaemia is mainly due to the dysfunction of the spleen, liver and heart. They are affected by factors such as eating habits, age and physique. A causal relationship exists between phlegm and blood stasis, which runs through the course of hyperlipidaemia. Therefore, the prevention and treatment of hyperlipidaemia from the perspective of phlegm and blood stasis treatment has a very broad prospect. As a TCM prescription, HQR possesses multiple cardioprotective effects against hyperlipidaemia and atherosclerosis.^11^ However, studies have rarely been reported on the effect on the myocardial protection provided by HQR after AMI in rats with obesity. HQR primarily includes *Salvia miltiorrhiza*, *Poria cocos*, *Acorus tatarinowii*, *Curcuma*, Rhizoma pinelliae preparatum, *Gynostemma*, *Codonopsis pilosula*, *Ligusticum chuanxiong Hort* and *Astragalus membranceus*. The chemical components of HQR—butane diacid, paeoniflorin, ferulic acid, astragaloside Ⅱ and astragaloside Ⅰ—were detected by HPLC‐Q‐TOF‐MS/MS (Figure 1). Treatment with paeoniflorin can reduce injury after AMI, and paeoniflorin plays cardioprotective roles by inhibiting iNOS signalling and inflammation.^34^ Paeoniflorin can also regulate hepatic cholesterol synthesis and metabolism, as well as improve blood lipid levels.^35^ Ferulic acid similarly improves blood lipid^36^ metabolism and can attenuate apoptosis caused by AMI by enhancing autophagy.^37^ Astragalosides can recover impaired ventricular function and prevent cardiac remodelling induced by hypercholesterolemia via antioxidative stress and regulation of cardiac homeostasis.^38^ They can also relieve myocardial injury in AMI rats via the upregulation of Notch1/Jagged1 signalling and hypoxia inducible factor‐1α.^39^ Ferulic acid plays cardioprotective roles by suppressing mitochondrial mitophagy dependence on PINK1/Parkin in H9c2 cells.^40^ Ferulic acid can improve mitochondrial dysfunction (increasing mPTP and decreasing MMP) in Alzheimer's disease.^41^ Astragalosides can decrease the reduction of ROS and release of Cyt‐c, reduce apoptosis, inactivate caspase‐3 and inhibit mPTP opening. Owing to such inhibition, astragalosides have been targeted as treatment.^42^ Currently, mitochondrial dysfunction (particularly the opening rate of mPTP) is suggested to perform an important function in aggravating damage after AMI,^43^ decreasing MMP, causing mitochondrial swelling and inhibiting oxidative phosphorylation via mPTP opening. The opening of the mPTP occurs via binding to the CyPD protein, which is expressed in the inner membrane of mitochondria. Previous studies about the heart have shown that CsA protects from IRI via binding to CyP, independent of anti‐calcineurin properties, thus suppressing mPTP opening.^44^ Thus, in the current study, we hypothesized that HQR can play a protective function on AMI via the treatment of mitochondrial dysfunction (inhibiting mPAP opening) and downregulation of inflammatory response.

Our study demonstrated that the rats fed with HFD for 8 weeks can overt obesity can be induced rats in the form of weight gain with an accumulation of perirenal fat. HFD can induce inflammation, hyperlipidaemia and oxidative stress (imbalance between oxidation and antioxidation). It can damage the mitochondrial dynamics/biogenesis and the respiratory chain complex enzyme in myocardial tissues in rats.^26^ In the present study, hyperlipidaemia can aggravate the damage to the myocardial histological structure in AMI, in contrast to non‐hyperlipidaemia. We found that HFD could induce obesity and hyperlipidaemia (increasing LDL‐C, TG and TC as well as reducing HDL‐C). Using HQR as a hypolipidaemic drug can improve hyperlipidaemia (Figure 2A). We used ELISA to examine IL‐1β and TNF‐α. The results indicated that AMI could induce systemic inflammatory response (elevated concentrations of TNF‐α and IL‐1β), which decreased when pretreated with HQR (Figure 2D). Different parts of myocardial tissues have different levels of collagen content, hence the variation in the effect of systemic inflammation.^45^

Acute myocardial infarction can induce myocardial injury (the infarct area and the non‐infarct area), exhibiting increases in the infarction size (%) and myocardial enzyme levels. Moreover, hyperlipidaemia can aggravate the myocardial damage induced by AMI (Figure 2C,E). HE staining of myocardial tissues in rats indicates that AMI can induce local necrosis, oedema and a disordered structure in myocardial cells (the non‐infarct area) (the effects are more apparent in the AMI group with obesity). However, pretreatment with HQR can reduce the injury of myocardial tissues, as indicated by an improvement of interstitial oedema and a decrease in the number of disordered cells (Figure 2G). Cardiac function can be reduced via the structural injury of the heart (Figure 2B). Meanwhile, the TUNEL assay revealed that AMI can promote myocardial apoptosis (Figure 3A). Nonetheless, HQR can decrease the number of apoptotic cells (Figure 3A). As the principal executors of apoptosis correlated with the final phase, caspase‐9/3 activity is a significant indication of apoptosis. Thus, we measured caspase‐9/3 activity in the present study. Caspase‐9/3 was highly activated in the AMI group, relative to that in the sham (particularly in the group with obesity) (*p* < 0.05). Meanwhile, the immunofluorescence results for the expression of the apoptosis‐related gene Bax and Bcl‐2 in the myocardial tissues revealed that AMI increased the Bax expression and decreased the Bcl‐2 expression, particularly in the obese group. Bax expression decreased, whereas Bcl‐2 expression increased after pretreatment with HQR (Figure 4).

AMI has previously been shown to induce mitochondrial dysfunction in myocardial tissue.^9^ Mitochondrial apoptotic signalling pathways (the ERK‐CREB and ERK1/2 pathways) play critical roles in AMI injury.^46^, ^47^ In the present study, AMI could induce an abnormal mitochondrial morphology (swelling and even membrane rupture) and further damaged the mitochondria; moreover, HQR could protect the mitochondria after AMI combined with hyperlipidaemia (Figure 5). Mitochondrial and redox homeostasis performs a key function in pathophysiology after AMI. An impaired mitochondrial function of myocardial cells and increasing oxidative stress are significant factors influencing AMI damage.^42^ Under conditions of ischaemia, lack of oxygen and substrates can repress mitochondrial respiration, and cells must translate to glycolysis, markedly decreasing the capability of cells to produce ATP rapidly. ATP depletion increases the osmotic gradient, which drives water into the mitochondrial matrix to promote cell swelling.^48^ In addition, hyperlipidaemia results in the mass production of mitochondrial ROS (hazardous substance), inducing mitochondrial oxidative injury, which activates caspase‐dependent podocyte apoptosis via the mitochondrial apoptotic signalling pathway, such as damaged MMP and mitochondrial respiratory function and induced apoptosis. Mitochondrial dysfunction results in myocardial injury.^49^, ^50^

In the current study, we built a model of AMI in rats with obesity pretreated with HQR. We determined the MMP, mPAP opening (%), ROS, mtDNA, oxygen consumption rate, RCR, the activity of mitochondrial respiratory chain complex enzymes (Ⅰ, Ⅱ, Ⅲ, Ⅳ and Ⅴ) and ATP, which were considered as indicators of mitochondrial function. RCR, MMP, oxygen consumption rate, mitochondrial respiratory chain complex enzymes (Ⅰ, Ⅱ, Ⅲ, Ⅳ and Ⅴ) and ATP were reduced in the AMI and AMI (obese) groups (particularly in the AMI [obese] group); however, the ROS and mPAP opening (%) were increased in the AMI group (markedly increased in the AMI [obese] group). Pretreatment with HQR could improve mitochondrial function (Figure 5).

The copy number of the mtDNA in each mitochondrion is constant; thus, the mtDNA total copy number can estimate the quantity of mitochondria.^21^, ^51^ Although the mechanism of mtDNA injury has yet to be determined, mtDNA is more fragile when exposed to oxidative reaction than the nucleus DNA, considering that mtDNA is near the respiratory chain. In the present study, we examined the damage of AMI on mtDNA (real‐time qPCR) by calculating the long/short fragment ratio (long/short ratio). The long/short fragment ratio was reduced by AMI, but pretreatment with HQR can increase the ratio (Figure 5).

As an important adaptation of exposure to chronic energy deprivation, mitochondrial biogenesis can occur in various complex factors, such as Tfam and Nrf1. Nrf1 can accelerate the transcription of nuclei‐encoded mitochondrial protein expression, including those that participate in oxidative phosphorylation and respiratory complexes. Tfam can enhance DNA replication and gene transcription of mitochondria by binding to the mitochondrial genome. PGC‐1α, an important transcriptional coactivator, can operate key elements such as Tfam and Nrf1 and together induce mitochondrial biogenesis.^52^ When the aforementioned genes are abnormally expressed, mitochondrial biogenesis becomes disorganized. As shown in Figure 6, AMI can substantially reduce the mRNA and protein expression of Nrf1, Tfam and PGC‐1α. HQR evidently increased the mRNA and protein expression of Nrf1, Tfam and PGC‐1α. After HQR intervention, an increased energy supply (ATP), decreased ROS and increased mitochondria biogenesis factors were observed. Together, these changes led to the increased shortage of intracellular energy supply. Harmful stimuli such as limited energy, ageing and oxidative stress can normally damage mitochondria, become enclosed in autophagosomes, fused to lysosomes and finally degraded.

Normally, mitochondria undergo a dynamic process, including fusion and fission. The dynamic course is important to preserve the shape, size and network of mitochondria. They are controlled by correlated proteins, including Drp1 and Mfns.^53^ The results of the present study (Figure 6) showed that the expression of Mfn1/2 and Drp1 was reversed after AMI, showing a chaotic mitochondrial fission–fusion balance. Mfn1/2 and Drp1 have important functions in mitochondrial fusion and fission, respectively. In this study, Mfn1/2 increased, whereas Drp1 decreased after AMI in non‐infarct tissue.

Activation of the PI3K/Akt/Bad signalling pathway function represses apoptosis mediated by mitochondria.^54^ The close relationship between them prompted us to evaluate the effect of PI3K. PI3K is a phosphatidylinositol kinase with activities similar to those of a serine/threonine‐specific protein kinase and a phosphatidylinositol kinase.^47^ After activation, the phosphatidylinositol family members on the cell membrane can be phosphorylated, and the downstream signal molecule Akt can be recruited and activated. The activated Akt then phosphorylates the Ser136/Ser112 residues of the Bad protein.^55^ Phosphorylated Bad is separated from the apoptosis‐promoting complex and forms a 14‐3‐3 protein complex, thereby inactivating its apoptosis‐promoting function, consequently inhibiting apoptosis.^56^ The present study showed that HQR effectively regulated the expression of apoptotic PI3K/Akt/Bad pathway‐related proteins; moreover, HQR could enhance PI3K and p‐Akt expression and downregulate the expression of Cyt‐c, cleaved caspase‐3 and PAR (Figure 7). These results showed that HQR could adjust the mitochondrial function and inhibit apoptosis induced by AMI via the activation of the PI3K/Akt/Bad pathway.

In the current study, isolated mitochondria were used, AMI promoted high ROS production, which damaged mtDNA. Ultimately, the mitochondrial respiratory function, biogenesis and dynamic functions deteriorated, leading to ROS generation, particularly in rats with obesity. Swelling of mitochondria was induced by mPTP opening after AMI. The mPTP opening induced proton flowback from the mitochondrial membrane space to the matrix. This process reduced ATP synthesis and MMP, resulting in metabolic abnormalities. MMP reduction, ATP synthesis and an increase in Cyt‐c were induced by the mPTP opening, leading to proton flowback from the mitochondrial membrane space to the matrix. Metabolic abnormalities were then induced, leading to apoptosis. However, pretreatment with HQR could inhibit apoptosis by the operating mitochondrial function via activation of the PI3K/Akt/Bad pathway in rats with obesity.

## AUTHOR CONTRIBUTIONS

**He Tai:** Data curation (lead); Investigation (lead); Methodology (lead); Software (lead); Writing – original draft (lead). **Yu‐jing Tong:** Data curation (lead); Formal analysis (lead); Investigation (lead); Writing – review & editing (equal). **Rui Yu:** Data curation (lead); Formal analysis (lead); Funding acquisition (lead); Writing – original draft (lead). **You Yu:** Data curation (equal); Formal analysis (equal); Investigation (equal); Software (equal); Supervision (equal); Writing – original draft (equal); Writing – review & editing (equal). **Si‐cheng Yao:** Data curation (equal); Formal analysis (equal); Investigation (equal); Methodology (equal). **Ling‐bing Li:** Conceptualization (equal); Data curation (equal); Formal analysis (equal); Investigation (equal); Methodology (equal); Project administration (equal); Writing – original draft (equal). **Ye Liu:** Conceptualization (equal); Data curation (equal); Formal analysis (equal); Investigation (equal). **Xiao‐zheng Cui:** Methodology (supporting); Supervision (supporting). **Jin‐song Kuang:** Methodology (lead); Validation (lead); Visualization (lead); Writing – review & editing (lead). **Xian‐sheng Meng:** Conceptualization (supporting); Investigation (supporting); Methodology (supporting); Writing – review & editing (lead). **Xiao‐lin Jiang:** Investigation (lead); Methodology (lead); Writing – review & editing (lead).

## CONFLICT OF INTEREST

The authors declare no conflict of interest.

## Data Availability

The datasets used and/or analysed during the current study are available from the corresponding author on reasonable request.
